# Triosephosphate isomerase 1 may be a risk predictor in laryngeal squamous cell carcinoma: a multi-centered study integrating bulk RNA, single-cell RNA, and protein immunohistochemistry

**DOI:** 10.1186/s40001-023-01568-8

**Published:** 2023-12-15

**Authors:** Jian-Di Li, Yi Chen, Shu-Wen Jing, Li-Ting Wang, Yu-Hong Zhou, Zhi-Su Liu, Chang Song, Da-Zhi Li, Hai-Quan Wang, Zhi-Guang Huang, Yi-Wu Dang, Gang Chen, Jia-Yuan Luo

**Affiliations:** 1 Department of Pathology, The First Affiliated Hospital of Guangxi Medical University, Guangxi Zhuang Autonomous Region, Shuangyong Road 6, Nanning, 530021 People’s Republic of China; 2 Guangxi Zhuang Autonomous Region Engineering Research Center for Artificial Intelligence Analysis of Multimodal Tumor Images, The First Affiliated Hospital of Guangxi Medical University, Guangxi Zhuang Autonomous Region, Shuangyong Road 6, Nanning, 530021 People’s Republic of China

**Keywords:** LSCC, TPI1, Clinicopathological significance, Tumor immune microenvironment, scRNA-seq, hdWGCNA

## Abstract

**Background:**

Although great progress has been made in anti-cancer therapy, the prognosis of laryngeal squamous cell carcinoma (LSCC) patients remains unsatisfied. Quantities of studies demonstrate that glycolytic reprograming is essential for the progression of cancers, where triosephosphate isomerase 1 (TPI1) serves as a catalytic enzyme. However, the clinicopathological significance and potential biological functions of TPI1 underlying LSCC remains obscure.

**Methods:**

We collected in-house 82 LSCC tissue specimens and 56 non-tumor tissue specimens. Tissue microarrays (TMA) and immunohistochemical (IHC) experiments were performed. External LSCC microarrays and bulk RNA sequencing data were integrated to evaluate the expression of TPI1. We used a log-rank test and the CIBERSORT algorithm to assess the prognostic value of TPI1 and its association with the LSCC microenvironment. Malignant laryngeal epithelial cells and immune-stromal cells were identified using inferCNV and CellTypist. We conducted a comprehensive analysis to elucidate the molecular functions of TPI1 in LSCC tissue and single cells using Pearson correlation analysis, high dimensional weighted gene co-expression analysis, gene set enrichment analysis, and clustered regularly interspaced short palindromic repeats (CRISPR) screen. We explored intercellular communication patterns between LSCC single cells and immune-stromal cells and predicted several therapeutic agents targeting TPI1.

**Results:**

Based on the in-house TMA and IHC analysis, TPI1 protein was found to have a strong positive expression in the nucleus of LSCC cells but only weakly positive activity in the cytoplasm of normal laryngeal cells (*p* < 0.0001). Further confirmation of elevated TPI1 mRNA expression was obtained from external datasets, comparing 251 LSCC tissue samples to 136 non-LSCC tissue samples (standardized mean difference = 1.06). The upregulated TPI1 mRNA demonstrated a high discriminative ability between LSCC and non-LSCC tissue (area under the curve = 0.91; sensitivity = 0.87; specificity = 0.79), suggesting its potential as a predictive marker for poor prognosis (*p* = 0.037). Lower infiltration abundance was found for plasma cells, naïve B cells, monocytes, and neutrophils in TPI-high expression LSCC tissue. Glycolysis and cell cycle were significantly enriched pathways for both LSCC tissue and single cells, where heat shock protein family B member 1, TPI1, and enolase 1 occupied a central position. Four outgoing communication patterns and two incoming communication patterns were identified from the intercellular communication networks. TPI1 was predicted as an oncogene in LSCC, with CRISPR scores less than -1 across 71.43% of the LSCC cell lines. TPI1 was positively correlated with the half maximal inhibitory concentration of gemcitabine and cladribine.

**Conclusions:**

TPI1 is dramatically overexpressed in LSCC than in normal tissue, and the high expression of TPI1 may promote LSCC deterioration through its metabolic and non-metabolic functions. This study contributes to advancing our knowledge of LSCC pathogenesis and may have implications for the development of targeted therapies in the future.

**Supplementary Information:**

The online version contains supplementary material available at 10.1186/s40001-023-01568-8.

## Background

Laryngeal cancer is a common malignant tumor of the respiratory system of origin. According to the latest cancer statistics report, there would be 184,615 new cases of laryngeal tumors and 99,840 deaths worldwide in 2020 [1]. In China, it is predicted that there will be 30,832 new cases of laryngeal tumors and 14,404 deaths in 2022 [2]. Moreover, laryngeal cancer severely affects the quality of life of patients [3], accompanied by impairment of speech, breathing, and swallowing ability. Therefore, both globally and in China, laryngeal cancer is featured by a high incidence, high mortality rate, and significant impact. The most common type of laryngeal cancer is laryngeal squamous cell carcinoma (LSCC). Some studies have shown that some common factors, such as dietary factors, environmental factors, and alcohol or tobacco abuse [4], are the major causal factors for the occurrence of LSCC [5]. Although great progress has been made in recent years in chemotherapy and radiotherapy, the prognosis of LSCC patients is still unsatisfactory, and there is an urgent need for clear molecular mechanisms related to the occurrence of LSCC to help improve the treatment and prognosis [6, 7]. However, numerous molecular mechanisms associated with LSCC remain unclear, specifically regarding the reprogramming of metabolism in LSCC. Further investigations are necessary to unveil the intricate mechanisms governing the development of LSCC [8–10].

In the context of cancer, including LSCC, a significant phenomenon known as the Warburg effect involves a metabolic reprogramming characterized by an increased reliance on glycolysis [11]. Of particular interest in this context is triosephosphate isomerase 1 (TPI1), an enzyme that plays a crucial role in glycolysis. TPI1 functions by catalyzing the conversion of dihydroxyacetone phosphate (DHAP) to D-type glyceraldehyde-3-phosphate (G3P) and vice versa. Remarkably, TPI1 is not only involved in metabolic processes [12, 13] but also contributes to tumorigenesis through non-metabolic mechanisms [14]. In terms of its metabolic influence, TPI1 has been implicated in promoting oral cancer [15], liver cancer occurrence [12, 13], and metastasis by modulating glycolytic levels within cancer cells. On the non-metabolic front, TPI1 has been found to enhance overall histone acetylation during the cell cycle [14], a critical factor in cancer-related cellular processes [16]. Despite these intriguing findings, the role of TPI1 in LSCC remains unexplored, which has captured our attention.

Herein, we studied the expression of TPI1 in LSCC using LSCC transcriptome data, in-house tissue microarrays (TMA), and related gene pathway analysis, and explored the possible mechanisms of TPI1 occurrence in LSCC single cells. All these results will contribute to a deeper understanding of the mechanisms of LSCC occurrence and have significant implications for the prevention, treatment, and improved prognosis of LSCC.

## Methods

### Data acquisition and processing of bulk RNA, immunohistochemistry, and single-cell RNA sequencing

#### Bulk RNA expression data of laryngeal squamous cell carcinoma tissue

Gene microarrays and RNA sequencing (RNA-seq) data of LSCC tissue worldwide were retrieved and downloaded using TCGA, GEO, SRA, and ArrayExpress databases, and the corresponding clinicopathological parameters were collected. The following search formula was used: ((laryngeal OR laryngeal squamous cell carcinoma OR LSCC OR HNSCC OR SCC OR carcinoma of larynx OR kehlkopfkrebs OR laryngopharynx) AND (tumor OR neoplasm OR phyma OR malignancy OR malignant OR malignance OR cancer OR carcinoma OR carcinosis)). The inclusion and exclusion criteria were: (1) inclusion of samples with both LSCC and normal tissue and exclusion of metastatic cancer tissue; (2) inclusion of datasets with sample size ≥ six and exclusion of duplicate samples; (3) inclusion of Homo sapiens samples and exclusion of animal samples. The datasets were merged according to the platform information, and abnormal data samples were removed, with intergroup correction followed by batch correction using the ComBat function of sva and limma-voom.

#### In-house tissue microarrays and immunohistochemistry of laryngeal squamous cell carcinoma

LSCC and non-tumor tissue samples were obtained from Pantomics, Co.Ltd to detect the expression level of TPI1 protein in LSCC. The enrolled tissue samples should meet the following conditions: (1) tumor samples were pathologically diagnosed as LSCC; (2) sufficient tumor and normal samples were dissected for protein immunohistochemical staining. A total of 82 in-house LSCC tissue specimens and 56 non-tumor tissue specimens were collected. These specimens were prepared using five TMA, specifically HNT961, HNT962, HNT1021, ORC1021, and TOC481. The protein expression activity of TPI1 in LSCC tissue was detected using two-step immunohistochemistry and semi-quantitative scoring methods. A slice without any antibody was used as the negative control group. The TMA experiment was authorized by the Ethics Committee of Pantomics, Co.Ltd.

#### Single-cell RNA sequencing profile of laryngeal squamous cell carcinoma

To gain a precise understanding of the expression activity and functional pattern of TPI1 in LSCC, we employed single-cell RNA-seq (scRNA-seq) analysis. A total of 4782 cells from LSCC samples (GSE213047, Illumina NextSeq 500) were included in our study [17]. The quality control (QC) metrics, as previously established by *Maoxuan Lin *et al. [17], were utilized to ensure the reliability and validity of the data. Following dimension reduction using a uniform manifold approximation and projection (UMAP) algorithm, single cells were effectively clustered with a resolution of 0.8. Cluster markers were identified using a Wilcoxon rank sum test, which were utilized to manually annotate the cell populations. In-depth cellular sub-population annotations were performed using scanpy and CellTypist [18] modules in Python environment, referring to the Immune_ALL_Low dataset. Annotation accuracy was further validated using SingleR. To account for large-scale chromosome copy number variation events, we employed inferCNV [19] to identify malignant epithelial cells within the dataset. Finally, we examined the expression distributions of TPI1 within the identified single-cell clusters.

### Evaluation of tumor immune microenvironment in laryngeal squamous cell carcinoma

#### Cell type identification by estimating relative subsets of RNA transcripts

The immune microenvironment of LSCC tissue was predicted using a deconvoluting algorithm called cell type identification by estimating relative subsets of RNA transcripts (CIBERSORT) [20]. According to the median expression value of TPI1 mRNA, LSCC patients were assigned to two groups, including TPI1-high expression group and TPI1-low expression group. The correlation between TPI1 expression and immune infiltration was quantitatively calculated in LSCC tissue.

#### Intercellular communication analysis between malignant epithelial cells and immune-stromal cells

To unravel the intricate interaction network between cancer cells and their surrounding microenvironment, we focus on investigating the communication dynamics among malignant epithelial cells, immune cells, and stromal cells within the LSCC single-cell profile using CellChat [21]. To predict cell state-specific communication, we identified overexpressed ligands or receptors in each cell population and then determined the overexpressed ligand-receptor interactions. The communication probability at the signaling pathway level was also calculated by aggregating the communication probabilities of all ligand-receptor interactions associated with each signaling pathway. Moreover, the outgoing communication patterns of secretory cells and the incoming communication mode of the target unit were identified using a non-negative matrix factorization clustering algorithm. This allowed us to gain insights into how different cell populations contribute to the overall communication landscape within the LSCC microenvironment.

#### Putative immune checkpoint therapy-associated gene signatures in laryngeal squamous cell carcinoma

To explore the potential association between TPI1 and immune checkpoint, we investigated the activity of PD-L1 expression and the PD-1 checkpoint pathway in LSCC single cells using AUCell [22]. We analyzed both wild-type cells and anti-PD1 non-responsive cells [17, 23] to gain insights into their immune checkpoint status. Using the AUCell_exploreThresholds function, each single cell was classified as either immune checkpoint activated or silent based on predetermined thresholds. This approach allowed us to examine the relationship between TPI1 expression and the activity of immune checkpoints in LSCC. By assessing differentially expressed genes between such two cell groups, we aimed to identify potential response indicators and therapeutic targets for immune therapy in LSCC patients.

### Prospective molecular mechanisms of triosephosphate isomerase 1 in laryngeal squamous cell carcinoma

#### Co-expression analysis of triosephosphate isomerase 1 in laryngeal squamous cell carcinoma tissue

Co-expressed genes of TPI1 were identified through Pearson correlation analysis in bulk LSCC tissue. If the Pearson correlation coefficient was ≥ 0.30, *p* < 0.05, and TPI1 was significantly positively correlated with a gene in at least three datasets, it could be indicated that the gene was potentially co-expressed with TPI1.

#### Differentially expressed genes in laryngeal squamous cell carcinoma tissue

The standardized mean difference (SMD) values were calculated for bulk RNA-seq data and gene microarrays of LSCC tissue to screen for highly expressed genes. The inclusion criteria for overexpressed genes were: (1) SMD > 0, and (2)* p* < 0.05.

#### Functional annotation of triosephosphate isomerase 1 co-expressed genes in laryngeal squamous cell carcinoma tissue

Highly expressed co-expression genes (HECEGs) of TPI1 were acquired by intersecting LSCC overexpressed genes and TPI1 co-expressed gene. To further explore the molecular functions of TPI1, gene ontology (GO) and Kyoto encyclopedia of genes and genomes (KEGG) pathway enrichment analyses were performed on the HECEGs of TPI1 in bulk LSCC tissue.

#### Single-cell weighted gene co-expression analysis

To investigate the potential molecular function of TPI1 in single cells of LSCC, we employed high dimensional weighted gene co-expression analysis (hdWGCNA) to identify highly aggregated co-expression module genes [24, 25]. Using the the TestSoftPowers function, we selected a soft-power threshold β that resulted in a preferable scale-free topological network. For each module gene set, we identified hub genes based on eigengene-based connectivity rankings. The biological functions of hub genes were annotated for each module using enrichR [26]. With a Seurat method, cell-specific gene function scores of were calculated. Transcriptional factor was predicted for each functional module by referring to the ChEA_2022 gene set. Finally, a protein-to-protein interaction network was established based on TPI1, the hub genes specific to LSCC, and the predicted transcriptional factor.

#### Preliminary verification for the molecular functions of triosephosphate isomerase 1

To elucidate the molecular roles of TPI1, we conducted a comprehensive analysis focused on both LSCC tissue and single cells. The hallmark gene sets, h.all.v2023.1.Hs.symbols.gmt, were first downloaded from the Molecular Signatures Database. Subsequently, we investigated the association between these predefined gene sets and the expression of TPI1 in LSCC tissue using a gene set enrichment analysis. Moreover, we assessed pathways activity and compared their relevance with TPI1 expression in LSCC single cells using AUCell. To further validate the biological functions of TPI1 in promoting LSCC cells, we performed a gene fitness effect analysis of TPI1 using clustered regularly interspaced short palindromic repeats (CRISPR) screen in seven LSCC cell lines available in DepMap [27].

### Putative therapeutic agents targeting triosephosphate isomerase 1

Based on the pro-tumor role TPI1 may play in LSCC, the druggable potential of TPI1 was assessed by integrating genomic and pharmacologic data in CellMiner [28]. Pearson correlation coefficient was calculated to determine the relevance between TPI1 expression and the half maximal inhibitory concentrations of 792 anti-cancer agents in a total of 59 cancer cell lines. Additionally, such cell lines were assigned to either TPI1-high expression group or TPI-low expression group. The half maximal inhibitory concentrations of 792 anti-cancer agents were compared between the two groups.

### Statistical methods

The statistical analysis was conducted using R v4.2.0. According to the design type in each dataset, the comparison of TPI1 expression between the two groups was assessed using a paired or unpaired Wilcoxon test. Additionally, normalized relative expression values of TPI1 was evaluated by calculating SMD values. If the SMD value was greater than zero, it indicated high expression of TPI1 in LSCC. The 95% confidence interval of SMD was considered statistically significant if it did not encompass zero. To assess the potential heterogeneity within the selected data, we employed the *I*^*2*^ statistic. When *I*^*2*^ ≤ *5*0% or *p* > 0.05, a fixed-effects model was utilized. Otherwise, a random-effects model was applied. Sensitive analysis was performed to investigate the possible reasons for heterogeneity. Receiver operating characteristic (ROC) curves and area under the curve (AUC) were generated using pROC. Summary ROC (SROC) curves were plotted, and sensitivity, specificity, positive likelihood ratio (PLR), and negative likelihood ratio (NLR) were calculated in STATA v12.0. To explore the prognostic value of TPI1 in LSCC patients, Kaplan–Meier analysis was conducted using data from TCGA, GSE65858, and GSE27020. The immune infiltration abundance between the TPI1-high expression group and TPI1-low expression group were compared using a Wilcoxon test. Significant differences were considered when *p* < 0.05.

## Results

### Clinicopathological significance of triosephosphate isomerase 1 overexpression in laryngeal squamous cell carcinoma

#### Comprehensive evaluation of triosephosphate isomerase 1 mRNA expression levels in global laryngeal squamous cell carcinoma and non-cancerous tissue

A total of 11 LSCC datasets were included in the present study (Additional file 1: Table S1). Based on the single-centered expression analysis results of gene chip and RNA-seq datasets (Additional file 1: Figures S1 and S2), it was found that in GPL6244 (*p* < 0.01, AUC = 0.7438) and GSE127165 (*p* < 0.0001, AUC = 0.8833), as well as in GSE51985 (*p* < 0.05, AUC = 0.84), GSE84957 (*p* < 0.01, AUC = 0.9506), GSE59102 (*p* < 0.001, AUC = 0.8276), and TCGA-LSCC (*p* < 0.0001, AUC = 0.8917), TPI1 was highly expressed in LSCC tissue as opposed to non-cancerous tissue.

**Fig. 1:**
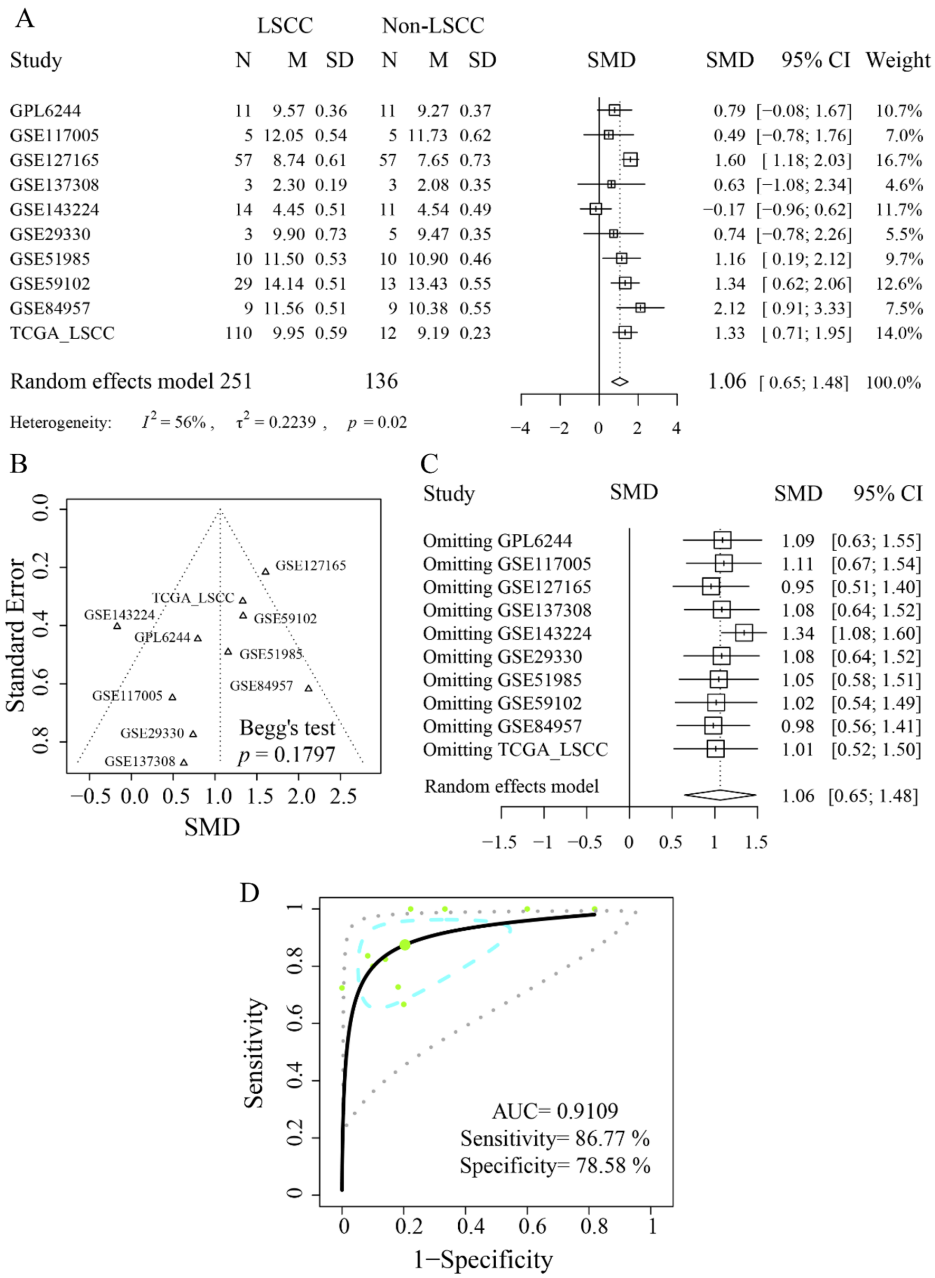
Integrated expression level of triosephosphate isomerase 1 mRNA in laryngeal squamous cell carcinoma tissue. The mRNA expression level of triosephosphate isomerase 1 (TPI1) was comprehensively calculated using quantitative synthesis. **A** Forest plot of TPI1 standardized mean difference in laryngeal squamous cell carcinoma tissue. **B** Funnel plot of publication bias detection (*p* = 0.9379). **C** Forest plot of sensitive analysis. Single dataset was omitted one by one to probe the potential source of heterogeneity. **D** A summary receiver operating characteristic curve with prediction and confidence contours

After quantitatively synthesis, it was observed that TPI1 mRNA expression showed a significant increase in 251 LSCC tissue samples compared to 136 non-LSCC tissue samples, despite the statistical heterogeneous among the included external datasets (Fig. 1A, *p* = 0.02 for the heterogeneity test, *I*^*2*^ = 56% > 50%, SMD = 1.06 [0.65, 1.48]). From the funnel plot in Fig. 1B, it can be seen that all the studies showed a relatively uniform distribution on both sides of the central axis (Begg’s rank correlation test of funnel plot asymmetry: *p* = 0.1797). When omitting each study one by one, the combined SMD effect sizes remain statistically significant, indicating that the stability of SMD result was not affected by inter-study heterogeneity (Fig. 1C).

Since TPI1 was markedly overexpressed in LSCC tissue, we further explored its ability to correctly identify LSCC cases and non-LSCC controls. With an AUC of 0.91, upregulated TPI1 showed a strong ability in differentiating LSCC from non-LSCC tissue (sensitivity = 0.87; specificity = 0.79) (Fig. 1D; Additional file 1: Figures S3A and B). Moreover, the PLR and NLR of discrimination were 4.72 and 0.17, indicating the relative accuracy of TPI1 in identifying LSCC from non-LSCC tissue (Additional file 1: Figures S3C and D).

#### Protein validation of triosephosphate isomerase 1 expression levels in laryngeal squamous cell carcinoma and normal tissue

**Fig. 2:**
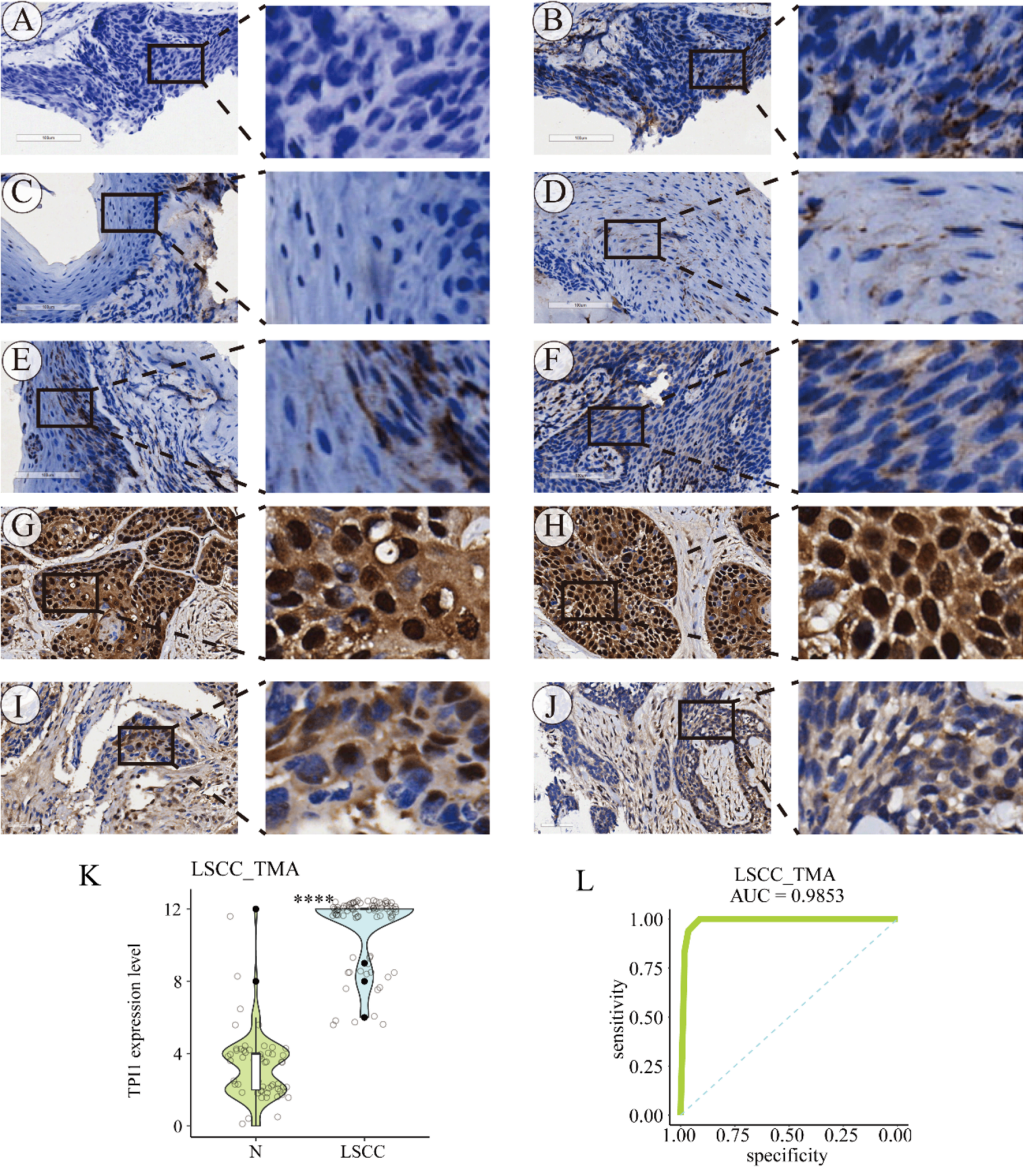
Protein expression level of triosephosphate isomerase 1 in laryngeal squamous cell carcinoma tissue. In-house tissue microarray and protein immunohistochemistry were performed to validate the expression trend of triosephosphate isomerase 1 (TPI1) in laryngeal squamous cell carcinoma (LSCC). Slice (**A**) was used as negative control, without using any antibody. Panels (**B**–**F**) and panels (**G**–**J**) presented the protein activity of TPI1 in non-LSCC and LSCC tissue, respectively. (**K**) Compared with non-LSCC tissue, LSCC tissue had a significantly high protein expression level of TPI1. **L** Over-expressed TPI1 showed a strong discriminatory ability between LSCC and non-LSCC tissue. ^****^, *p* < 0.0001

The immunohistochemical activity of TPI1 protein was probed in non-LSCC and LSCC tissue samples. TPI1 protein was weakly positive in the cytoplasm of normal laryngeal cells. Notably, when compared with the negative control (Fig. 2A) and non-LSCC cells (Fig. 2B–F), LSCC cells showed a strong positive expression of TPI1 protein in the nucleus and cytoplasm (Fig. 2G–J), which was statistically significant (Fig. 2K). Furthermore, overexpressed TPI1 protein showed a strong discriminatory ability between LSCC and non-LSCC tissue (AUC = 0.9853) (Fig. 2L).

#### Sub-population distribution of triosephosphate isomerase 1 expression in laryngeal squamous cell carcinoma single cells

**Fig. 3:**
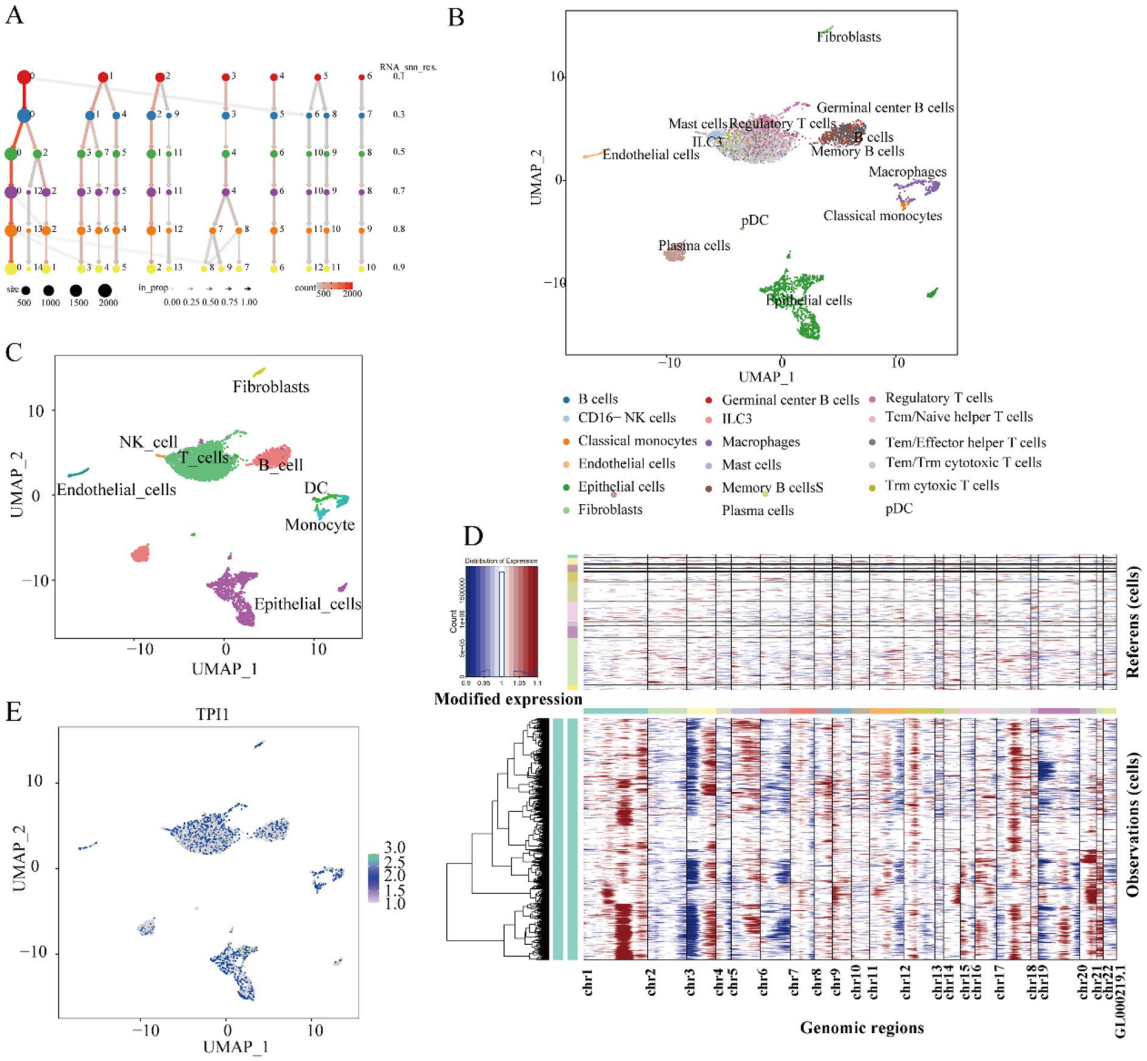
Single-cell analysis of laryngeal squamous cell carcinoma. The potential molecular mechanisms of anti-PD-1-resistant laryngeal squamous cell carcinoma (LSCC) was dissected form the perspective of single cells. **A** The clustering stability was checked at the given resolution. **B** Tem/Trm cytotoxic T cells (25.37%) were identified as the predominant cell types in LSCC lesions, followed by epithelial cells (20.62%) and regulatory T cells (10.83%). **C** Single cells isolated from the LSCC tissue sample were also annotated using singleR, in addition to CellTypist. **D** Epithelial cells were highly malignant compared with the reference cells, covering T cells, B cells, CD16 (−) natural killer cells, classical monocytes, macrophages, endothelial cells, and fibroblasts. **E** Triosephosphate isomerase 1 was widely expressed in malignant epithelial cells and immune cells

The expression distributions of TPI1 were explored in the LSCC single cells. QC violin plots confirmed that mitochondrial genes in the analyzed LSCC single cells were under 20% (Additional file 1: Figure S4). When exploiting a resolution at 0.80, the UMAP clustering results of LSCC single cells were relatively stable (Fig. 3A). According to the machine learning annotation results of CellTypist, Tem/Trm cytotoxic T cells (25.37%) were identified as the predominant cell types in LSCC lesions, followed by epithelial cells (20.62%) and regulatory T cells (10.83%) (Fig. 3B). The annotated results were generally consistent with that of singleR and manual annotation (Fig. 3C and Additional file 1: Figure S5). Epithelial cells were highly malignant compared with the reference cells, covering T cells, B cells, CD16 (−) natural killer cells, classical monocytes, macrophages, endothelial cells, and fibroblasts (Fig. 3D). TPI1 was widely expressed in malignant epithelial cells and immune cells (Fig. 3E).

#### Prognostic assessment of triosephosphate isomerase 1 in different laryngeal squamous cell cancer cohorts

**Fig. 4:**
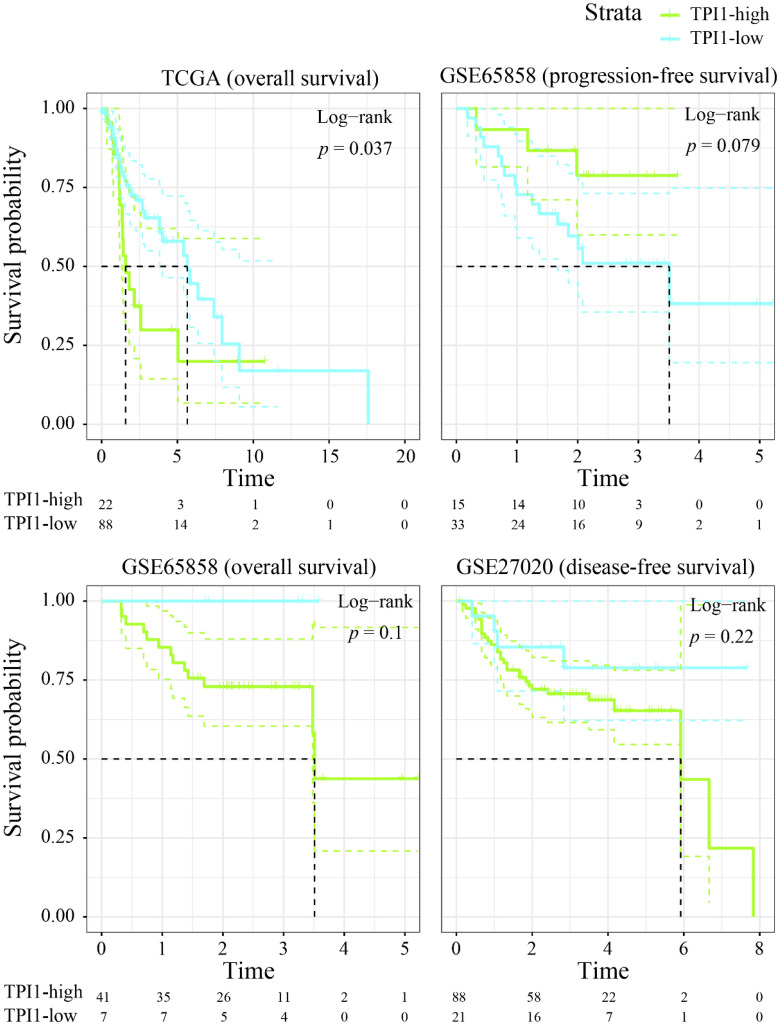
Prognostic value of triosephosphate isomerase 1 in laryngeal squamous cell carcinoma. Overexpressed triosephosphate isomerase 1 may be a significant risk factor for the overall survival of laryngeal squamous cell carcinoma patients

According to the Kaplan–Meier survival curves, highly expressed TPI1 mRNA predicted poor overall survival condition in the TCGA-LSCC prognostic cohort (Fig. 4, *p* = 0.037). However, the prognostic ability of TPI1 was insignificant when evaluating the prognosis of LSCC patients in GSE65858 and GSE27020 (Fig. 4).

### Potential role of triosephosphate isomerase 1 in tumor microenvironment in laryngeal squamous cell carcinoma

#### Correlation between triosephosphate isomerase 1 overexpression and tumor microenvironment in laryngeal squamous cell carcinoma tissue

**Fig. 5:**
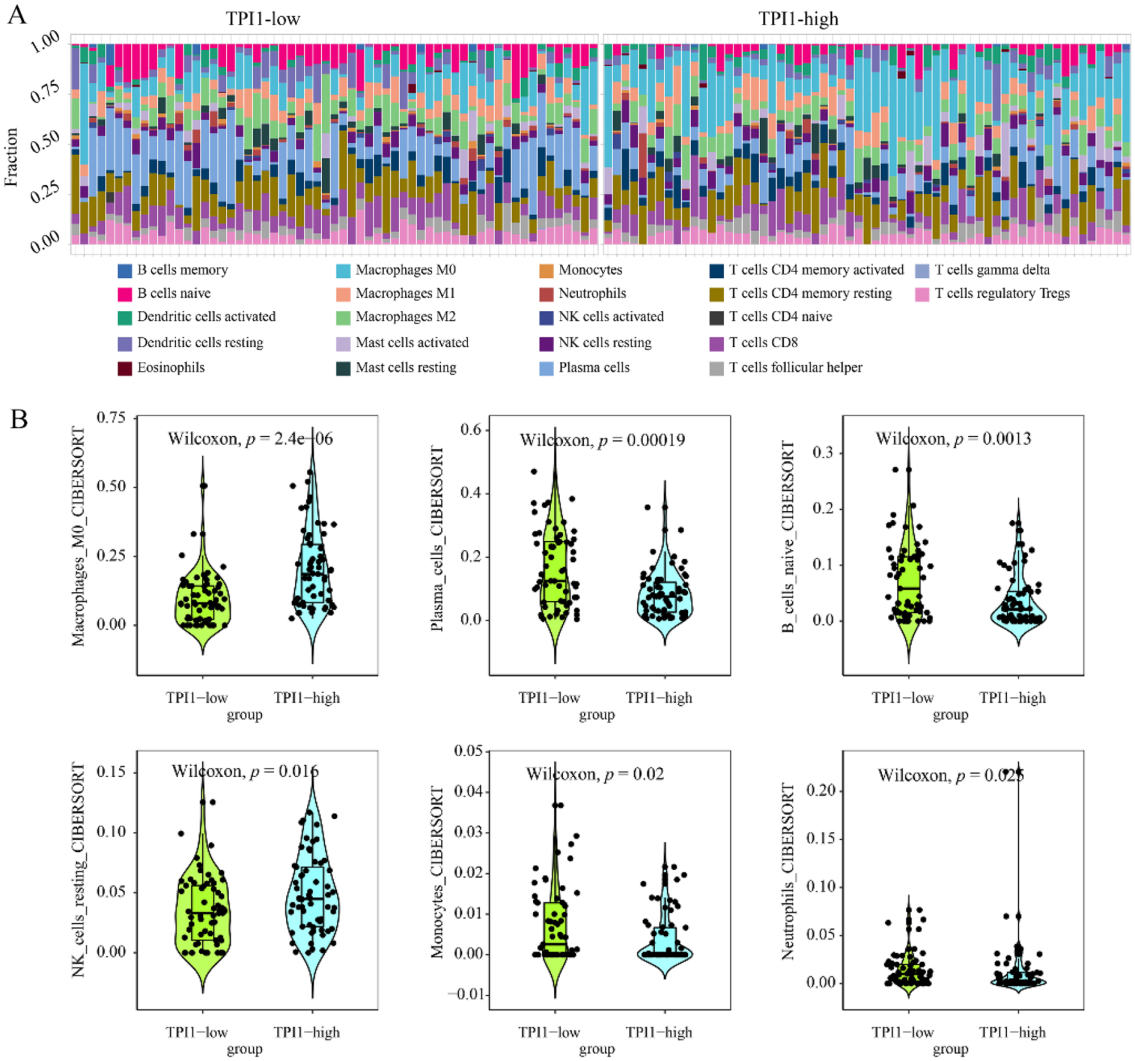
Immune correlation between triosephosphate isomerase 1 and immune infiltrating cells in laryngeal squamous cell carcinoma. **A** The tumor microenvironment composition was compared between triosephosphate isomerase 1 (TPI1) high expression group and TPI1 low expression group. **B** Compared with TPI1-low expression group, TPI1-high expression group showed a significant higher infiltration levels of M0 macrophages and resting natural killer cells. Additionally, TPI1-high expression group showed lower infiltration levels of plasma cells, naïve B cells, monocytes, and neutrophils

The immune infiltration abundance was visualized in TPI1-low expression group and TPI1-high expression group (Fig. 5A). Compared with TPI1-low expression group, TPI1-high expression group showed a significant higher infiltration levels of M0 macrophages and resting natural killer cells (Fig. 5B). However, TPI-high expression group showed lower infiltration levels of plasma cells, naïve B cells, monocytes, and neutrophils. Additionally, the expression levels of TPI1 mRNA were positively correlated to the infiltration levels of M0 macrophages (R = 0.398; *p* = 5.55E-06) but negatively correlated to that of plasma cells (R = −0.352; *p* = 6.97E-05) and naïve B cells (R = −0.344; *p* = 0.0001).

#### Intercellular communication network of laryngeal squamous cell carcinoma cells and immune-stromal cells

**Fig. 6:**
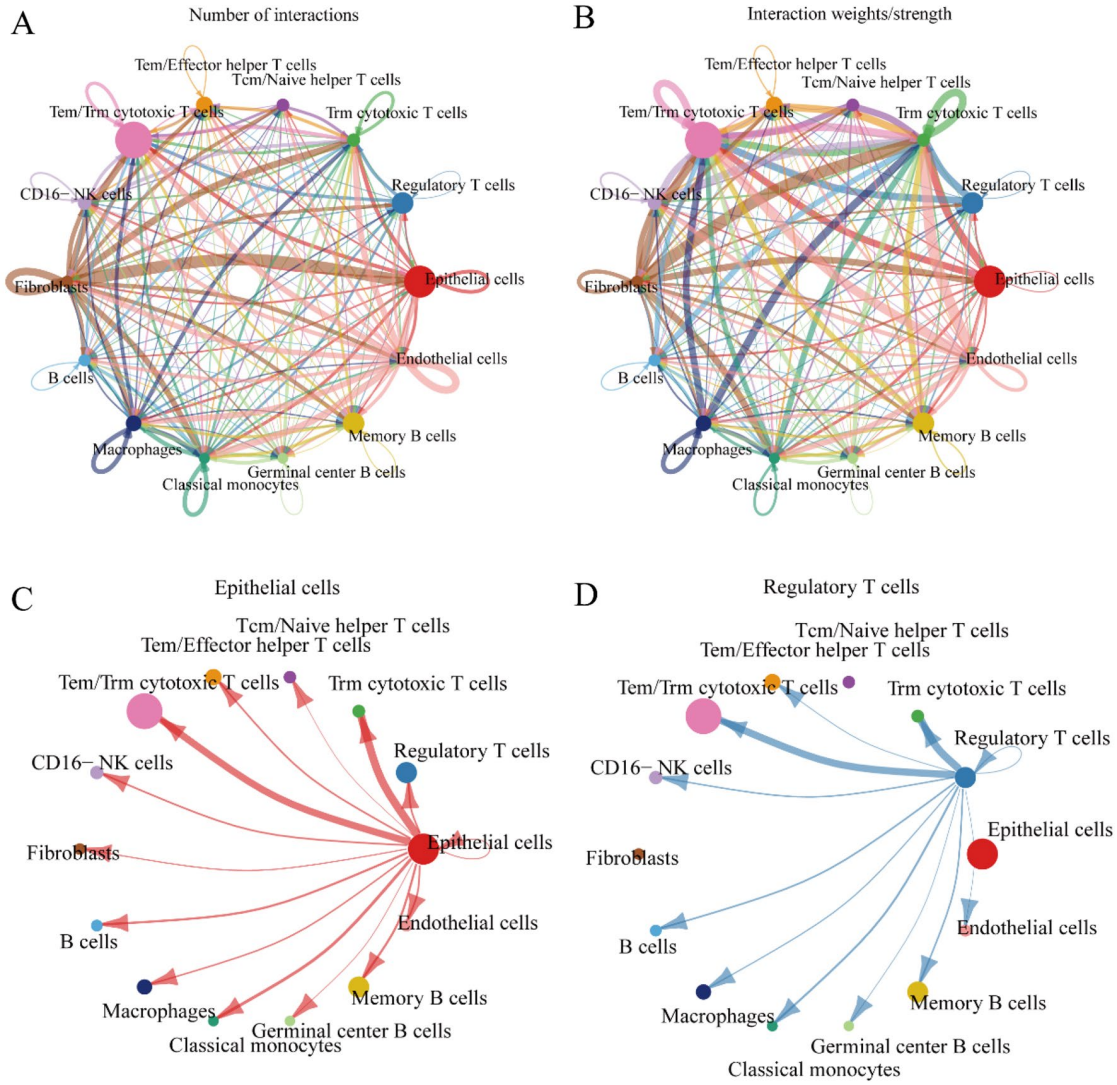
Intercellular communication in laryngeal squamous cell carcinoma. We explored the intercellular communication networks between malignant epithelial cells and immune-stromal cells in laryngeal squamous cell carcinoma by calculating **A** the number of communications and **B** the probability of communications. Circles of different colors represented different cell populations, the size of the circle was proportional to the number of cells corresponding to the cell populations, the color of each edge was consistent with the signal sender, and the thickness of the edge was proportional to the number and strength of intercellular communication. Panel (**C**, **D**) presented the communication probability of malignant epithelial cells and regulatory T cells

**Fig. 7:**
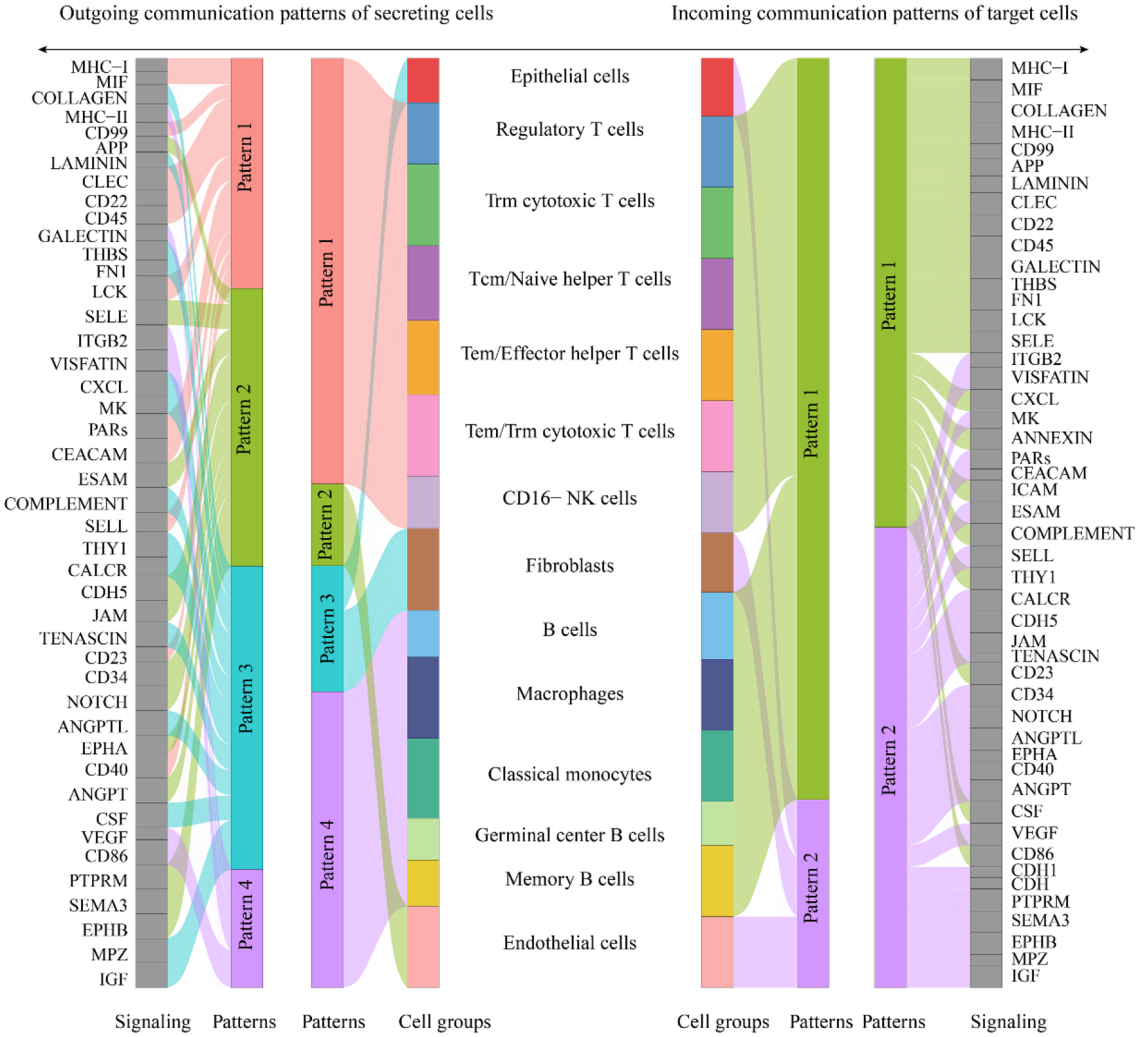
CellChat analysis of cell-to-cell communication in laryngeal squamous cell carcinoma. We identified the global communication patterns of malignant epithelial cells and immune-stromal cells in laryngeal squamous cell carcinoma. Both outgoing communication patterns of secretory cells and incoming communication patterns of target cells were visualized to explore how multiple cell types and signaling pathways work together

The intricated communicating networks between malignant epithelial cells and immune-stromal cells were explored in LSCC (Fig. 6A–B), including paracrine signaling, extracellular matrix-receptor signaling, and cell–cell contact. A total of 181 important ligand-receptor pairs were detected in 18 cell populations, which were furtherly classified into 48 signaling pathways, including CEACAM, MHC-I, MHC-II, NOTCH, VEGF, etc. As the predominant cell group in LSCC single cells, cytotoxic T cells were identified as the prominent signal receiver of malignant epithelial cells and regulatory T cells (Fig. 6C–D). Moreover, four outgoing communication patterns and two incoming communication patterns were identified from the intercellular communication networks of LSCC (Fig. 7). For the outgoing communication, malignant epithelial cells and fibroblasts were involved in the pattern three. In this pattern, the major signaling contributor covered COLLAGEN, LAMININ, THBS, FN1, CXCL, MK, COMPLEMENT, THY1, CALCR, TENASCIN, ANGPTL, CSF, MPZ, and IGF.

#### Identification of immune checkpoint therapy-associated gene signatures in laryngeal squamous cell carcinoma single cells

We assessed the activity of PD-L1 expression and PD-1 checkpoint pathway (hsa05235) in wild type and anti-PD1 non-responsive LSCC single cells (Additional file 1: Figure S6A, B). However, TPI1’s expression level showed no significant differences between the immune checkpoint activated and silent groups (data not shown). We identified 821 differentially expressed genes between the immune checkpoint activated and silent groups, which were notably enriched in pathways related to Th1 and Th2 cell differentiation, as well as PD-L1 expression and the PD-1 checkpoint pathway (Additional file 1: Figure S6C, D).

### Prospective molecular mechanisms of triosephosphate isomerase 1 in laryngeal squamous cell carcinoma

#### Co-expression network of triosephosphate isomerase 1 in laryngeal squamous cell carcinoma bulk samples

A total of 285 TPI1 HECEGs were screened out from the bulk LSCC tissue specimens. In terms of GO categories, TPI1 HECEGs were mainly involved in mitotic nuclear division, nuclear division, single-stranded DNA binding, and DNA polymerase binding (Additional file 1: Figure S7). Moreover, TPI1 HECEGs were significantly enriched in the signaling cascades of cell cycle, DNA replication, nucleotide excision repair, p53 signaling pathway, etc*.* in addition to glycolysis/gluconeogenesis.

#### High dimensional weighted gene co-expression network of triosephosphate isomerase 1 in laryngeal squamous cell carcinoma single-cell samples

**Fig. 8:**
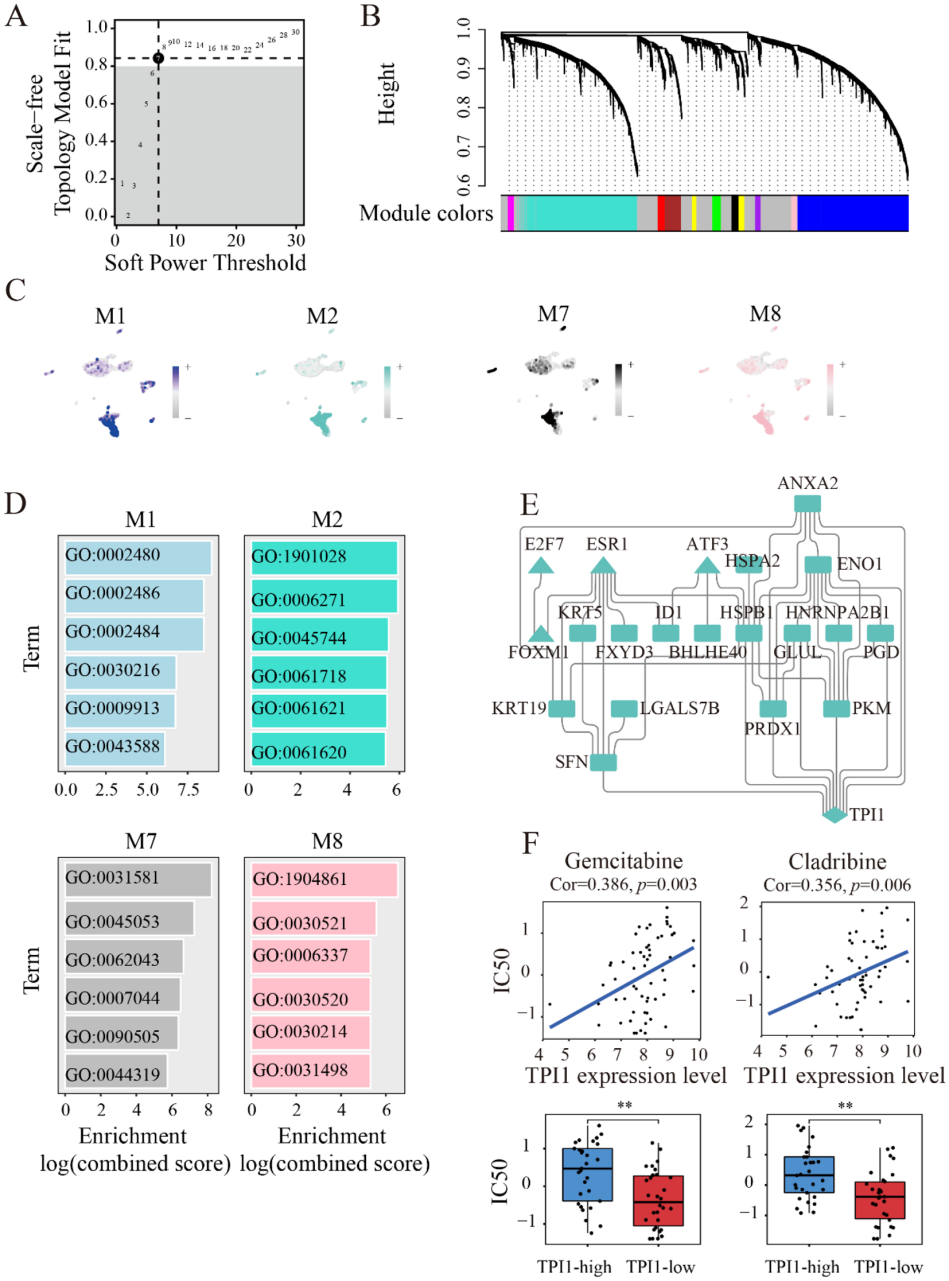
Potential biological functions of co-expression gene modules in laryngeal squamous cell carcinoma single cells. **A** Soft power threshold selection for single-cell weighted gene co-expression analysis of laryngeal squamous cell carcinoma. **B** Malignant epithelial cells were subset to conduct high dimensional weighted gene co-expression analysis. **C** According to the gene function scores, four co-expression gene modules were found relatively laryngeal squamous cell carcinoma (LSCC)-specific. **D** The biological processes mapped to four gene modules specific to LSCC. **E** Protein interaction network of M2 gene module and triosephosphate isomerase 1 (TPI1) in LSCC. Triangle refers to the transcription factor and rectangle refers to the common protein. To explore the therapeutic potential of TPI1, cellminer analysis was performed on the genomic and pharmacologic data. Higher expression level of TPI1 could be used to predict higher half maximal inhibitory concentration of gemcitabine and cladribine (**F**)

The co-expression mechanisms underlying LSCC were further explored using hdWGCNA. A threshold of soft power for hdWGCNA was set as *β* = 9, slightly higher than the minimum scale-free topology model fit index in LSCC single cells (Fig. 8A). Ten gene modules were identified from the malignant epithelial cells (Fig. 8B). Among them, four co-expression gene modules were relatively specific to the malignant epithelial cells (Fig. 8C; Additional file 1: Figure S8). Interestingly, the M2 module was functionally related to TPI1 by mediating glycolysis/gluconeogenesis and cell cycle pathways. The M2 module were significantly enriched in glucose catabolic process to pyruvate (GO:0061718), canonical glycolysis (GO:0061621), and glycolytic process through glucose-6-phosphate (GO:0061620) (Fig. 8D). Therefore, we dived into the protein interaction network of M2 gene module and TPI1, as well as their transcriptional factor, covering E2 factor transcription factor 7 (E2F7), estrogen receptor 1 (ESR1), and forkhead box M1 (FOXM1) (Fig. 8E). Finally, heat shock protein family B member 1 (HSPB1), TPI1, and enolase 1 (ENO1) occupied a central position for the function of M2 module.

#### Potential oncogene roles of triosephosphate isomerase 1 in laryngeal squamous cell carcinoma

**Fig. 9:**
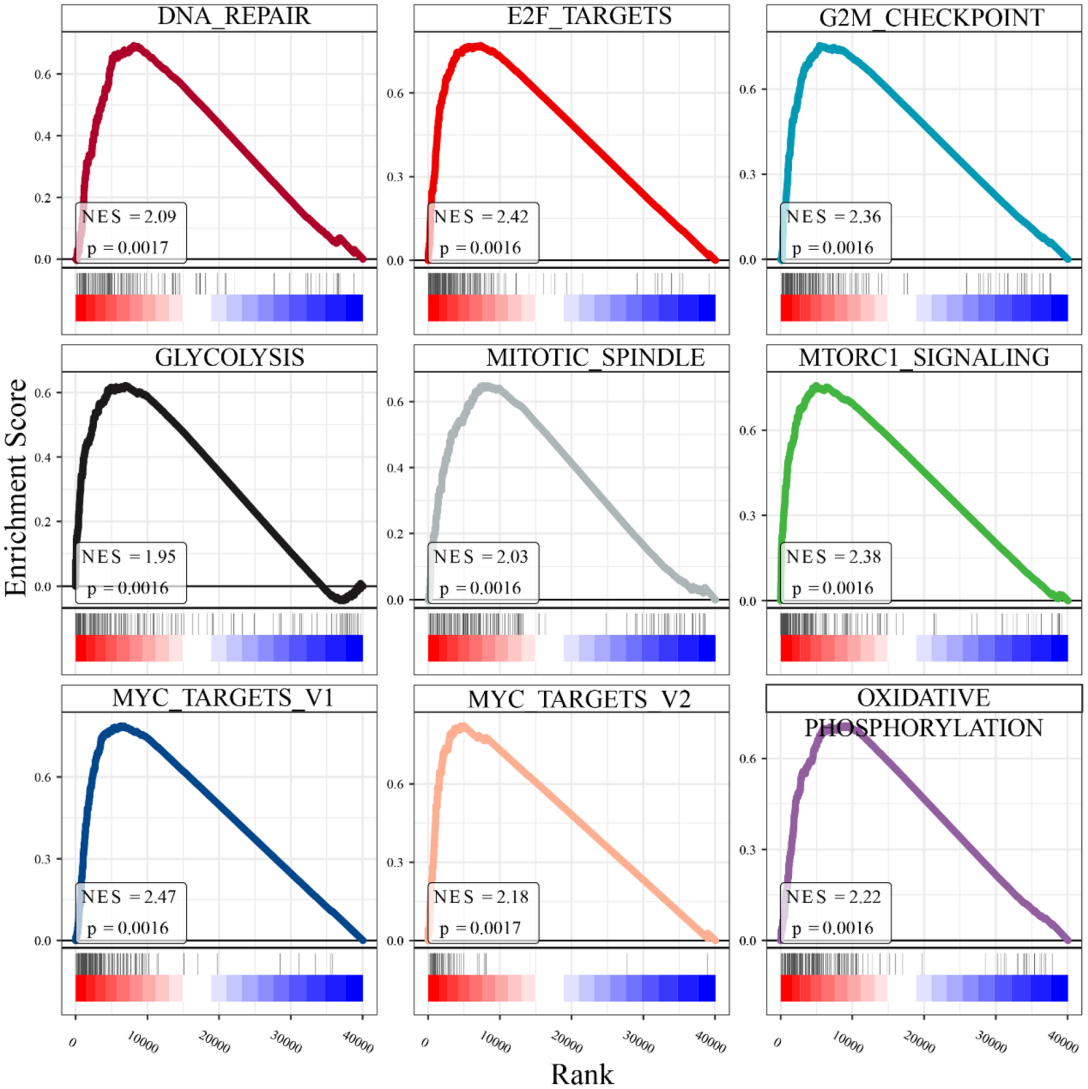
Gene set enrichment analyses of triosephosphate isomerase 1 in laryngeal squamous cell carcinoma tissue. Genes were ranked by the expression levels of triosephosphate isomerase 1 (TPI1). Significant correlations were observed between TPI1 expression and increased activities of the cell cycle and glycolysis processes. These findings suggest that TPI1 may play a crucial role in regulating the cell cycle and promoting glycolysis in laryngeal squamous cell carcinoma tissue

**Fig. 10:**
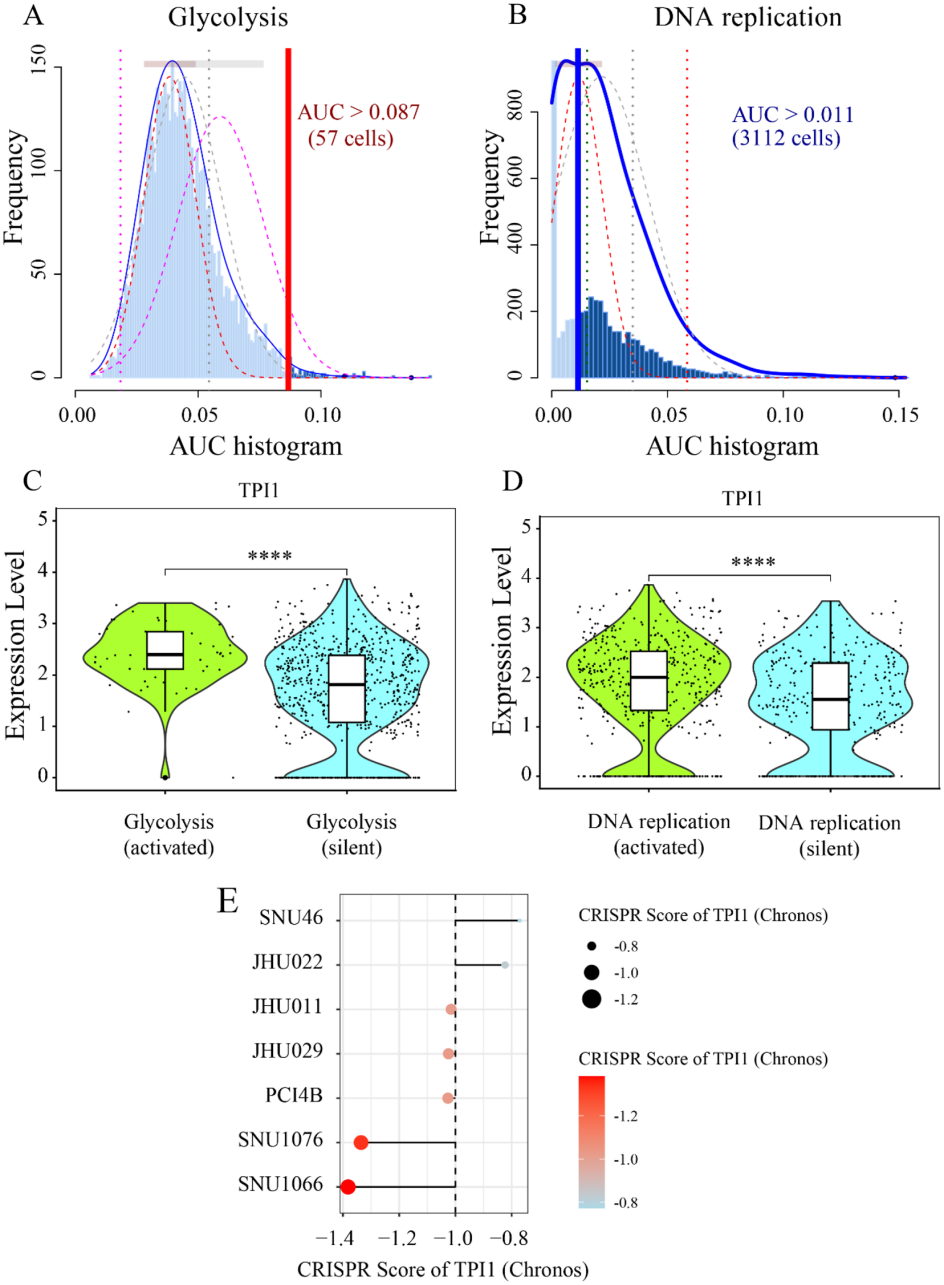
Potential pro-tumor roles of triosephosphate isomerase 1 in laryngeal squamous cell carcinoma single cells. **A**, **B** Glycolysis and DNA replication activities were calculated in laryngeal squamous cell carcinoma (LSCC) single cells using AUCell. **C**, **D** Analysis revealed a strong positive correlation between the expression levels of triosephosphate isomerase 1 (TPI1) and both glycolysis and DNA replication activities. **E** Gene fitness effect analysis of TPI1 in LSCC cell lines using clustered regularly interspaced short palindromic repeats (CRISPR) screen. These findings suggest that TPI1 was essential for the survival and proliferation of LSCC cells and elevated TPI1 expression may contribute to enhanced glycolysis and DNA replication processes in LSCC single cells. ^****^, *p* < 0.0001

In LSCC tissue, TPI1 overexpression was associated with increased enrichment in glycolysis and cell cycle related pathways, including DNA repair, E2F targets, G2M checkpoint, mitotic spindle, mitotic signaling, and MYC targets (Fig. 9). Similarly, higher TPI1 expression in LSCC single cells correlated with elevated activities in glycolysis and DNA replication (Fig. 10A–D). Notably, TPI1 was predicted as an oncogene in LSCC, with CRISPR scores below −1 across 71.43% of the analyzed LSCC cell lines (Fig. 10E).

### Potential therapeutic agents targeting triosephosphate isomerase 1 in laryngeal squamous cell carcinoma

Given the potential oncogenic role of TPI1 in LSCC, we explored the potential of TPI1-targeted drug development. The expression level of TPI1 mRNA was significantly correlated with the half maximal inhibitory concentration of gemcitabine and cladribine. Additionally, higher expression level of TPI1 could be used to predict higher half maximal inhibitory concentration of gemcitabine and cladribine (Fig. 8F).

## Discussion

It is well known that the development of many cancers is associated with metabolism reprograming at the cellular level [29]. As a common initial pathway for both aerobic and anaerobic oxidation, glycolysis is indeed associated with the development of many cancers [30]. In the process of glycolytic reprograming, TPI1, one of the key enzymes, have been shown to be closely associated with the deterioration of many cancers, such as intrahepatic cholangiocarcinoma [31, 32], pancreatic cancer [33], breast cancer [34], and lung cancer [35, 36]. However, abnormal TPI1 levels have never been reported to be associated with LSCC. In the present study, the comprehensive patterns of TPI1 overexpression were confirmed using worldwide bulk RNA data and intra-group protein immunohistochemistry, as well as scRNA-seq result, which reflects the strong evidence-based philosophy throughout our study. Furthermore, we probed the intricate co-expression network of TPI1 in promoting glycolysis and cell cycle in both LSCC tissue and single cells. Our study provides important clues for depicting the molecular landscape of TPI1 underlying LSCC.

TPI1 activity was significantly enhanced in LSCC tissue and single cells at the mRNA and protein levels. Based on the evidence-based concept, worldwide gene microarrays and RNA-seq datasets were collected to complete the quantitatively expression analysis of TPI1 mRNA in LSCC tissue. Although the results were statistically heterogeneous, the results showed the overexpression of TPI1 in 251 LSCC tissue samples, as is opposed to 136 non-LSCC tissue samples, and the plausibility was confirmed by SROC curves. More importantly, compared with cytoplasm localization in normal laryngeal cells, TPI1 protein was observed to be accumulated in the nucleus of LSCC cells. Surprisingly, it was reported that the translocation of TPI1 to cell nucleus could induce the chemoresistance of lung adenocarcinoma cells [37]. Herein, a translocation from cytoplasm to nucleus was also observed for TPI1 protein in LSCC cells. Moreover, higher mRNA expression levels of TPI1 were predicted to produce poorer survival outcomes in LSCC patients. Taken together, it is conceivable that the elevated activity and nuclear translocation of TPI1 may promote the occurrence and progression of LSCC.

Furthermore, the complicated molecular mechanisms of TPI1 underlying LSCC were addressed using both bulk RNA and scRNA-seq analysis. It is noted that glycolysis and cell cycle pathways were significantly enriched in both LSCC tissue and single cells. In the present study, we not only identified a LSCC-specific gene co-expression module from the hdWGCNA network, but also determined the putative transcriptional regulators and hub genes of them.

As is well known, glycolysis is regarded as a hallmark of cancers. Head and neck squamous cell carcinoma exhibited strong mitochondrial and glycolytic reserving ability [38, 39]. To support the huge energy consumption, head and neck cancer cells become highly glycolytic [40]. This makes glycolysis the predominant source coupled with oxidative phosphorylation, which promotes the metastasis of head and neck cancer cells. In the cascade reaction of glycolysis, TPI1 enzyme catalyzes the isomerization of DHAP and G3P. Additionally, DHAP can also be converted into glycerol 3-phosphate, which is a pivotal substance linking glucose metabolism and fat metabolism [41]. Large amounts of studies have reported that enhanced glycolysis or glycolytic reprograming could result in the invasion and chemoresistance of LSCC cells [10, 42, 43]. For instance, upregulated glycolysis was correlated to the progression and immune escape in head and neck squamous cell carcinoma [44]. In-depth investigation demonstrated that glycolysis and viability were promoted in LSCC cells by mediating the miR-377-3p/lactate dehydrogenase A axis [45]. Based on such findings, glycolysis blockage provides a possibility for treating squamous cell carcinoma of the head and neck [46]. Surprisingly, the inhibition of TPI1 and glucose-6-phosphate isomerase was demonstrated to attenuate the proliferation and invasion ability of MDA-MB-231 cells [47]. Taken together, it was reasonable to propose that upregulated TPI1 may facilitates the development of LSCC cells by enhancing glycolysis.

Uncontrollable cell cycle progression acts as a trigger for the development of cancers [48]. Herein, our study put novel insights into the association between TPI1 and cell cycle. The co-expression gene set of TPI1 was identified in LSCC tissue and single cells, which were consistently mapped to the cell cycle pathway. More excitingly, for the intercellular communication network of LSCC single cells, the malignant laryngeal epithelial cells and fibroblasts indeed sent out an outgoing communication signal by coordinating with the MK signaling pathway, which was considered to be required for cell cycle progression [49–51]. Although there is no report showing the precise functional mechanisms of TPI1 in the cell cycle of LSCC cells, few studies have demonstrated the association between them in other solid tumors. For example, cyclin-dependent kinase 2 could phosphorylate TPI1 Ser 117 and promote the nuclear translocation of TPI1 [14]. Given the enhanced nuclear accumulation of TPI1 protein in the LSCC cells, we could speculate that the cell-cycle-related genes co-expressed with TPI1 may promote the translocation of TPI1 protein and facilitate its activity in glycolytic reprograming. Additionally, TPI1 promotes the progression of breast cancer cells by regulating cell division cycle proteins and activating phosphoinositide 3-kinase/AKT serine/threonine kinase 1/mammalian target of rapamycin pathway [52]. Highly expressed TPI1 and the other glycolytic regulators were also suggested to be involved in cell cycle of prostate cancer [53]. When the glycolytic module (contains TPI1) is inhibited, there is a decrease in the S phase and an increase in the G2/M phase of the cell cycle and a decrease in the aggressiveness of hepatocellular carcinoma [54]. Moreover, TPI1 downregulation inhibits DNA replication in human fibroblasts and others and delays the entry of cells into the S phase ^(55)^. Therefore, it is suggested that there may be a bidirectional promotion mechanism between TPI1 and the cell cycle of malignant laryngeal squamous epithelial cells, which ultimately triggers LSCC progression. Further studies are needed to determine how TPI1 regulates the cell cycle, cell division, and other approaches to promote the mechanism of LSCC.

However, our limitations are also obvious. For example, although overexpression of TPI1 predicts poor survival outcome in the TCGA-LSCC cohort, its prognostic value in the other three cohorts was either insignificant or, in some cases, contradictory. Two factors may contribute to these discrepancies. Firstly, there were several confounding factors that could have influenced the prognosis of LSCC patients. For example, in the GSE65858 cohort, variations in treatment strategies, human papillomavirus status, and clinical stages were observed among different patients. Secondly, the limited sample size in these cohorts might have compromised the representativeness and reliability of the prognostic value of TPI1. Further validation of the prognostic value of TPI1 in LSCC is warranted in larger cohorts. Additionally, the molecular biological functions of TPI1 in LSCC have not been experimentally confirmed. The possible role that TPI1 plays in the communication between LSCC cells and immune-stromal cells is less known. Functional experiments must be carried out to certify the finding of the present study. Despite that, the results of our work reveal the upregulation of TPI1 at both the mRNA and protein levels. More importantly, we emphasized the important role TPI1 plays in promoting glycolysis and cell cycle of LSCC single cells, which will enrich our understanding of the occurrence and progression of LSCC. We expect further studies in the future to fill in our shortcomings.

## Conclusions

TPI1 is dramatically highly expressed in LSCC than in normal tissue, and the high expression of TPI1 may promote LSCC deterioration through its metabolic and non-metabolic functions, which will facilitate our future understanding of the detailed mechanisms of TPI1 in the development of LSCC.

### Supplementary Information

**Additional file 1: Table S1.** Laryngeal squamous cell carcinoma data sets included in the present study. **Figure S1.** mRNA expression levels of triosephosphate isomerase 1 in laryngeal squamous cell carcinoma tissue and non-cancerous tissue. In single data sets, the mRNA levels of triosephosphate isomerase 1 were increased in laryngeal squamous cell carcinoma tissue samples when compared with non-cancerous tissue samples. LSCC, laryngeal squamous cell carcinoma. **Figure S2.** Sensitivity and specificity of triosephosphate isomerase 1 overexpression in laryngeal squamous cell carcinoma tissue. AUC, area under the curve. **Figure S3.** Potential distinguished ability of triosephosphate isomerase 1 in laryngeal squamous cell carcinoma tissue. (A, B) Highly expressed triosephosphate isomerase 1 (TPI1) displayed a strong discriminatory ability between laryngeal squamous cell carcinoma tissue and non-cancerous tissue specimens, with high degrees of sensitivity and specificity. (C, D) The positive likelihood ratio (PLR) and negative likelihood ratio (NLR) also reflected the discriminatory accuracy of TPI1. **Figure S4.** Quality control for the single-cell RNA sequencing analysis of laryngeal squamous cell carcinoma. Cells with mitochondrial genes less than 20 % were preserved. **Figure S5.** Single-cell annotation analysis of laryngeal squamous cell carcinoma. Single cells isolated from the laryngeal squamous cell carcinoma tissue sample were also annotated by using classical cell markers, in addition to CellTypist. **Figure S6.** Putative immune checkpoint therapy-associated gene signatures in laryngeal squamous cell carcinoma single cells. (A, B) The activity of PD-L1 expression and PD-1 checkpoint pathway were calculated in wild type and anti-PD1 non-responsive laryngeal squamous cell carcinoma (LSCC) single cells using AUCell. (C) Potential gene signatures associated with immune checkpoint therapy were identified from LSCC single cells. (D) Kyoto encyclopedia of genes and genomes pathway enrichment analysis of such signatures. These findings provide preliminary evidence regarding putative immune checkpoint therapy-associated gene signatures in LSCC single cells, suggesting potential targets and pathways that could be explored for therapeutic interventions. **Figure S7.** Putative molecular mechanisms of triosephosphate isomerase 1 co-expressed genes in laryngeal squamous cell carcinoma tissue. **Figure S8.** Hub genes of laryngeal squamous cell carcinoma specific co-expressed gene modules. A total of four laryngeal squamous cell carcinoma specific gene modules were identified, including M1, M2, M7, and M8. Hub genes were determined by calculating the eigengene-based connectivity of each module gene.

## Data Availability

The datasets used and analyzed during the current study are available from the corresponding author on reasonable request.

## Abbreviations

LSCC: Laryngeal squamous cell carcinoma
TPI1: Triosephosphate isomerase 1
DHAP: Dihydroxyacetone phosphate
G3P: D-type glyceraldehyde-3-phosphate
RNA-seq: RNA sequencing
SMD: Standardized mean difference
ROC: Receiver operating characteristic
AUC: Area under the curve
SROC: Summary receiver operating characteristic
PLR: Positive likelihood ratio
NLR: Negative likelihood ratio
scRNA-seq: Single-cell RNA sequencing
QC: Quality control
UMAP: Uniform manifold approximation and projection
CIBERSORT: Cell type identification by estimating relative subsets of RNA transcripts
HECEGs: Highly expressed co-expression genes
GO: Gene Ontology
KEGG: Kyoto encyclopedia of genes and genomes
hdWGCNA: High dimensional weighted gene co-expression analysis
HSPB1: Heat shock protein family B member 1
ENO1: Enolase 1
