# Supplementary material for: Triosephosphate isomerase 1 may be a risk predictor in laryngeal squamous cell carcinoma: a multi-centered study integrating bulk RNA, single-cell RNA, and protein immunohistochemistry
Source: Eur J Med Res. 2023 Dec 15;28:591. doi: 10.1186/s40001-023-01568-8 (PMC10724924; doi:10.1186/s40001-023-01568-8)
Supplement: Supplementary file 1 — Additional file 1: Table S1. Laryngeal squamous cell carcinoma data sets included in the present study. Figure S1. mRNA expression levels of triosephosphate isomerase 1 in laryngeal squamous cell carcinoma tissue and non-cancerous tissue. In single data sets, the mRNA levels of triosephosphate isomerase 1 were increased in laryngeal squamous cell carcinoma tissue samples when compared with non-cancerous tissue samples. LSCC, laryngeal squamous cell carcinoma. Figure S2. Sensitivity and specificity of triosephosphate isomerase 1 overexpression in laryngeal squamous cell carcinoma tissue. AUC, area under the curve. Figure S3. Potential distinguished ability of triosephosphate isomerase 1 in laryngeal squamous cell carcinoma tissue. (A, B) Highly expressed triosephosphate isomerase 1 (TPI1) displayed a strong discriminatory ability between laryngeal squamous cell carcinoma tissue and non-cancerous tissue specimens, with high degrees of sensitivity and specificity. (C, D) The positive likelihood ratio (PLR) and negative likelihood ratio (NLR) also reflected the discriminatory accuracy of TPI1. Figure S4. Quality control for the single-cell RNA sequencing analysis of laryngeal squamous cell carcinoma. Cells with mitochondrial genes less than 20 % were preserved. Figure S5. Single-cell annotation analysis of laryngeal squamous cell carcinoma. Single cells isolated from the laryngeal squamous cell carcinoma tissue sample were also annotated by using classical cell markers, in addition to CellTypist. Figure S6. Putative immune checkpoint therapy-associated gene signatures in laryngeal squamous cell carcinoma single cells. (A, B) The activity of PD-L1 expression and PD-1 checkpoint pathway were calculated in wild type and anti-PD1 non-responsive laryngeal squamous cell carcinoma (LSCC) single cells using AUCell. (C) Potential gene signatures associated with immune checkpoint therapy were identified from LSCC single cells. (D) Kyoto encyclopedia of genes and genomes pathway e [file 40001_2023_1568_MOESM1_ESM.docx]

# Additional file 1

Table S1: Laryngeal squamous cell carcinoma data sets included in the present study

| Dataset | Platform | Non-LSCC | LSCC | Study design |
| --- | --- | --- | --- | --- |
| GSE51985 | GPL10558 | 10 | 10 | Paired |
| GSE137308 | GPL16791 | 3 | 3 | paired |
| GSE84957 | GPL17843 | 9 | 9 | paired |
| GSE117005 | GPL20115 | 5 | 5 | paired |
| GSE127165 | GPL20301 | 57 | 57 | paired |
| GSE58911 | GPL6244 | 7 | 7 | paired |
| GSE107591 | GPL6244 | 4 | 4 | paired |
| GSE143224 | GPL5175 | 11 | 14 | Unpaired |
| GSE29330 | GPL570 | 5 | 3 | Unpaired |
| GSE59102 | GPL6480 | 13 | 29 | Unpaired |
| TCGA-LSCC | / | 12 | 110 | Unpaired |

Note: LSCC, laryngeal squamous cell carcinoma.
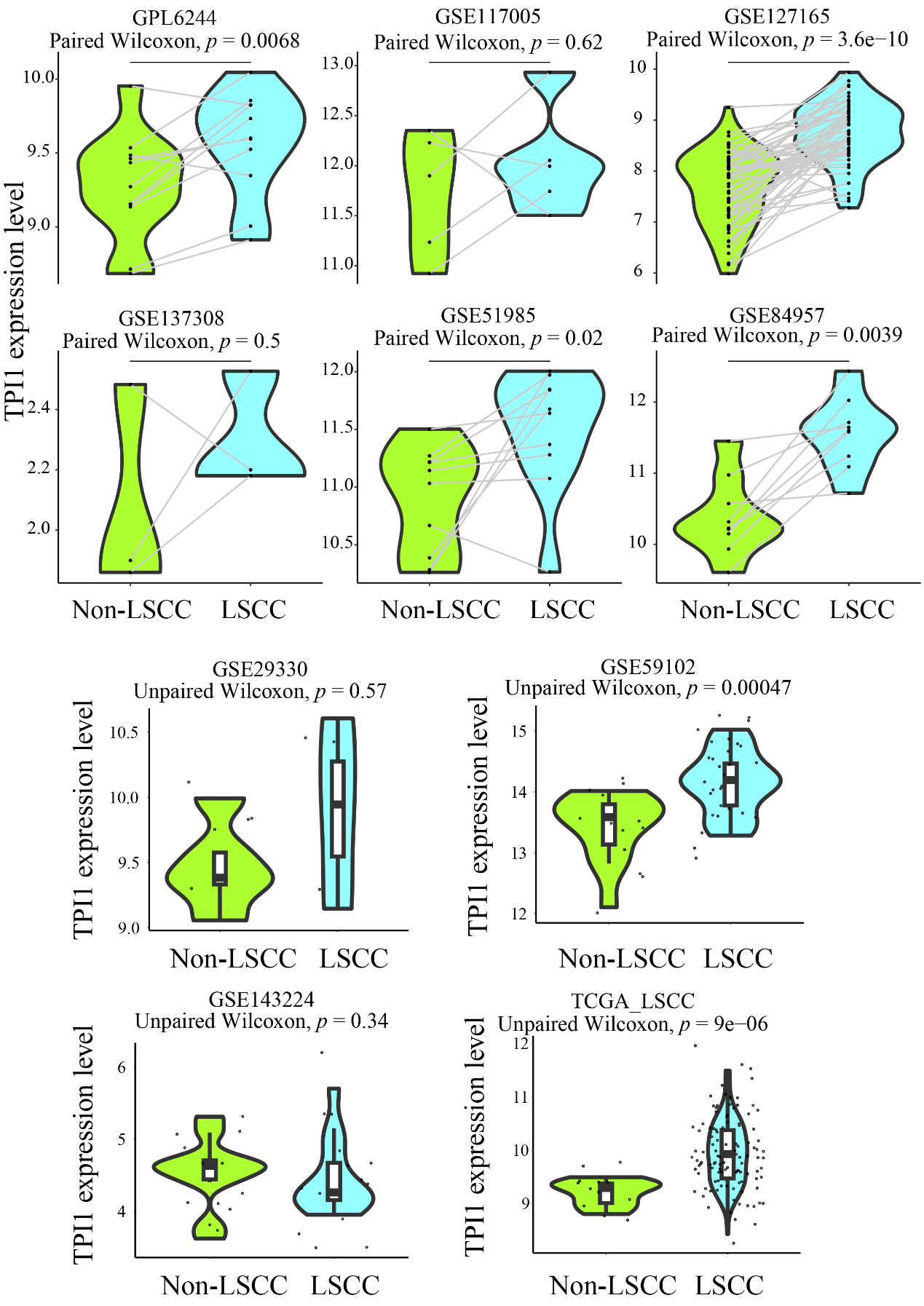


Figure S1: mRNA expression levels of triosephosphate isomerase 1 in laryngeal squamous cell carcinoma tissue and non-cancerous tissue

In single data sets, the mRNA levels of triosephosphate isomerase 1 were increased in laryngeal squamous cell carcinoma tissue samples when compared with non-cancerous tissue samples. LSCC, laryngeal squamous cell carcinoma.


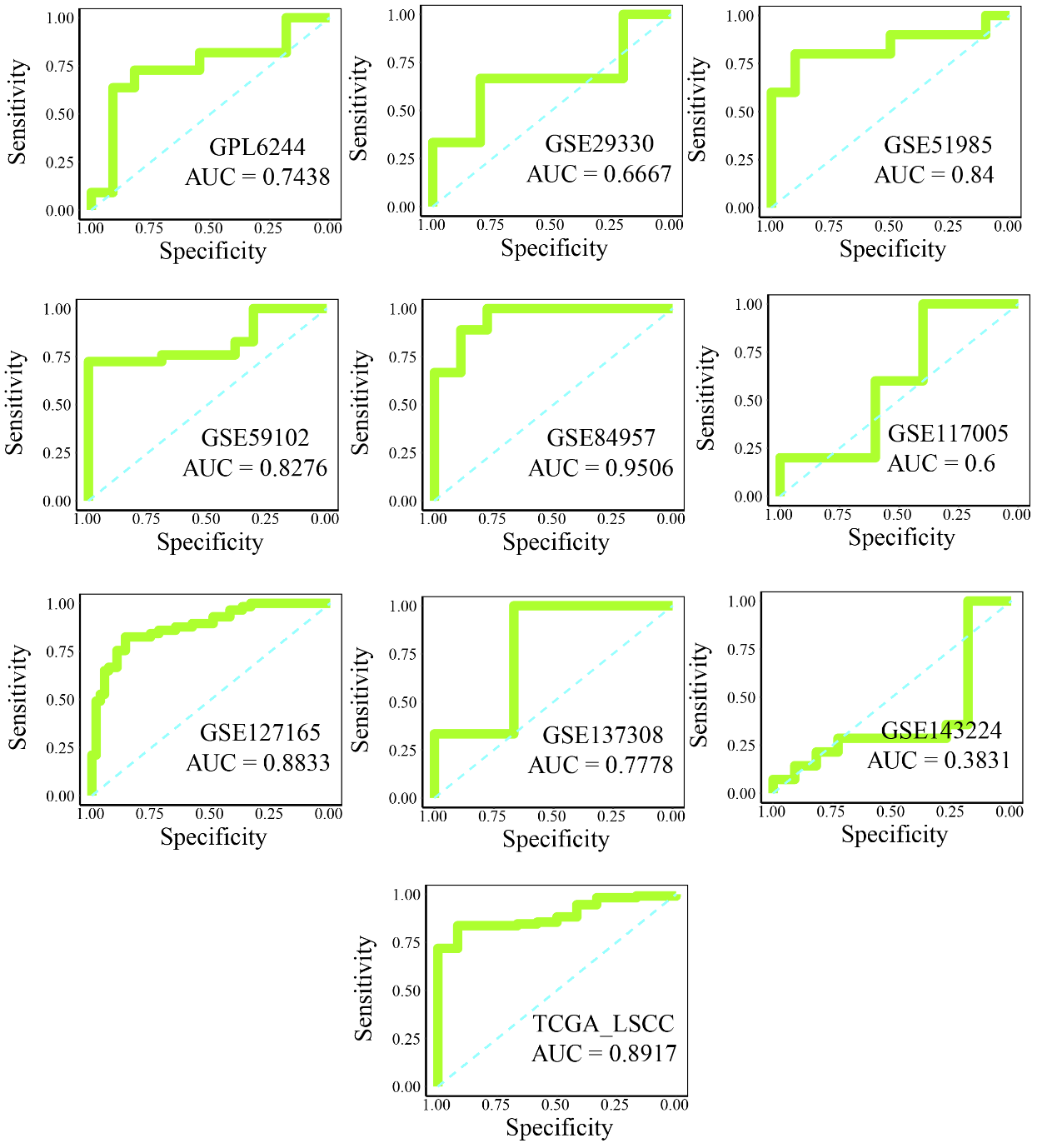
 Figure S2: Sensitivity and specificity of triosephosphate isomerase 1 overexpression in laryngeal squamous cell carcinoma tissue

AUC, area under the curve.


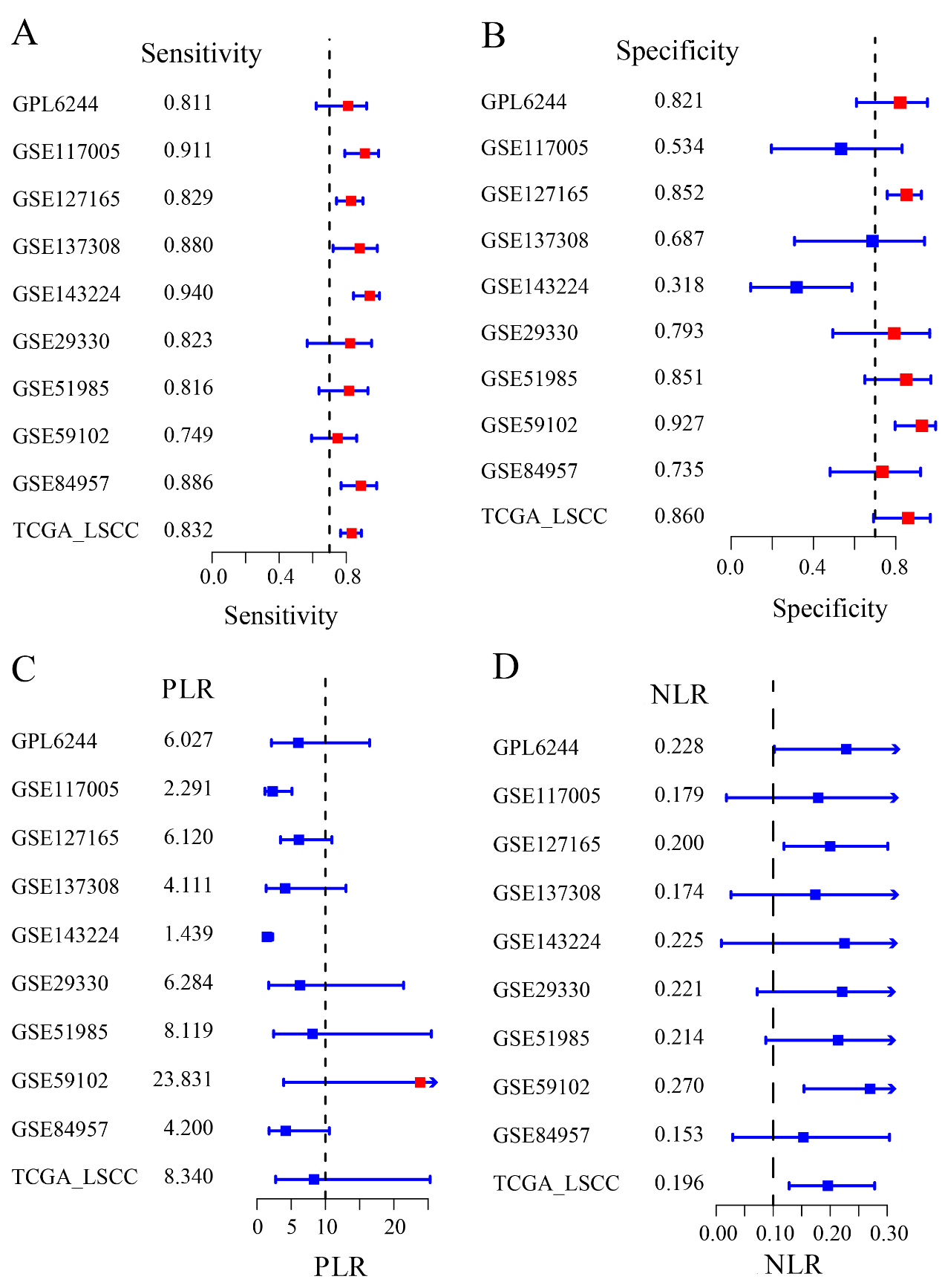


Figure S3: Potential distinguished ability of triosephosphate isomerase 1 in laryngeal squamous cell carcinoma tissue

(A, B) Highly expressed triosephosphate isomerase 1 (TPI1) displayed a strong discriminatory ability between laryngeal squamous cell carcinoma tissue and non-cancerous tissue specimens, with high degrees of sensitivity and specificity. (C, D) The positive likelihood ratio and negative likelihood ratio also reflected the discriminatory accuracy of TPI1. PLR, positive likelihood ratio; NLR, negative likelihood ratio.


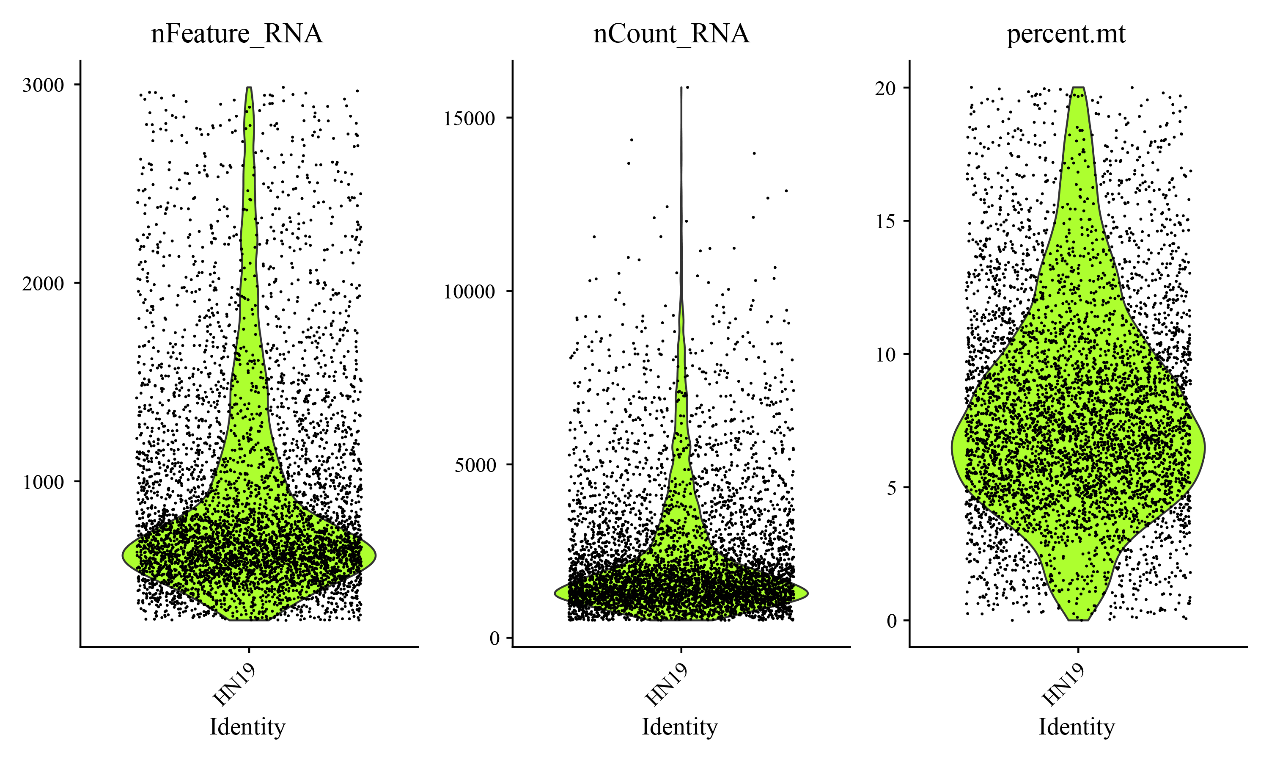


Figure S4: Quality control for the single-cell RNA sequencing analysis of laryngeal squamous cell carcinoma

Cells with mitochondrial genes less than 20 % were preserved.


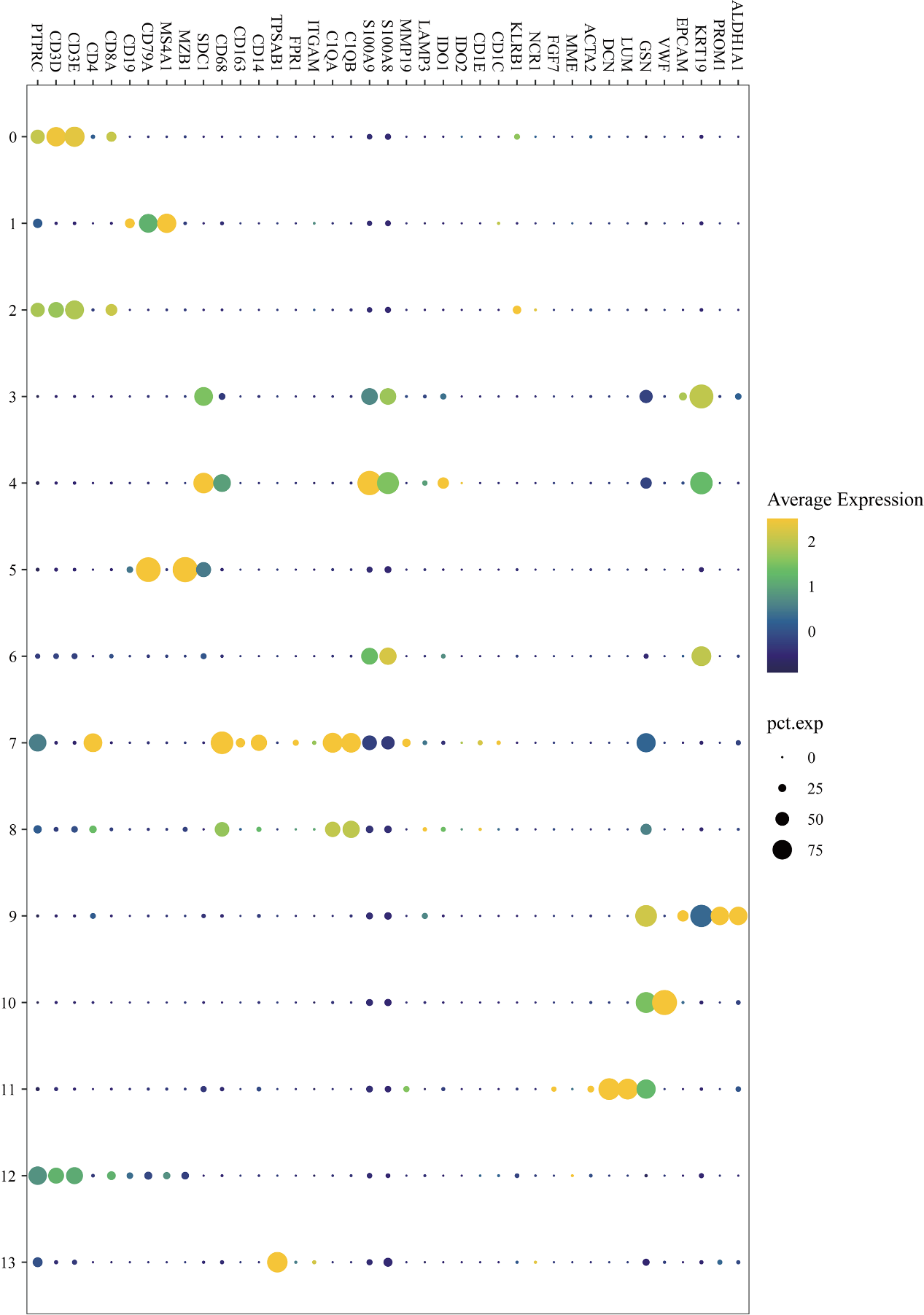


Figure S5: Single-cell annotation analysis of laryngeal squamous cell carcinoma

Single cells isolated from the laryngeal squamous cell carcinoma tissue sample were also annotated by using classical cell markers, in addition to CellTypist.

were determined by calculating the eigengene-based connectivity of each module gene.


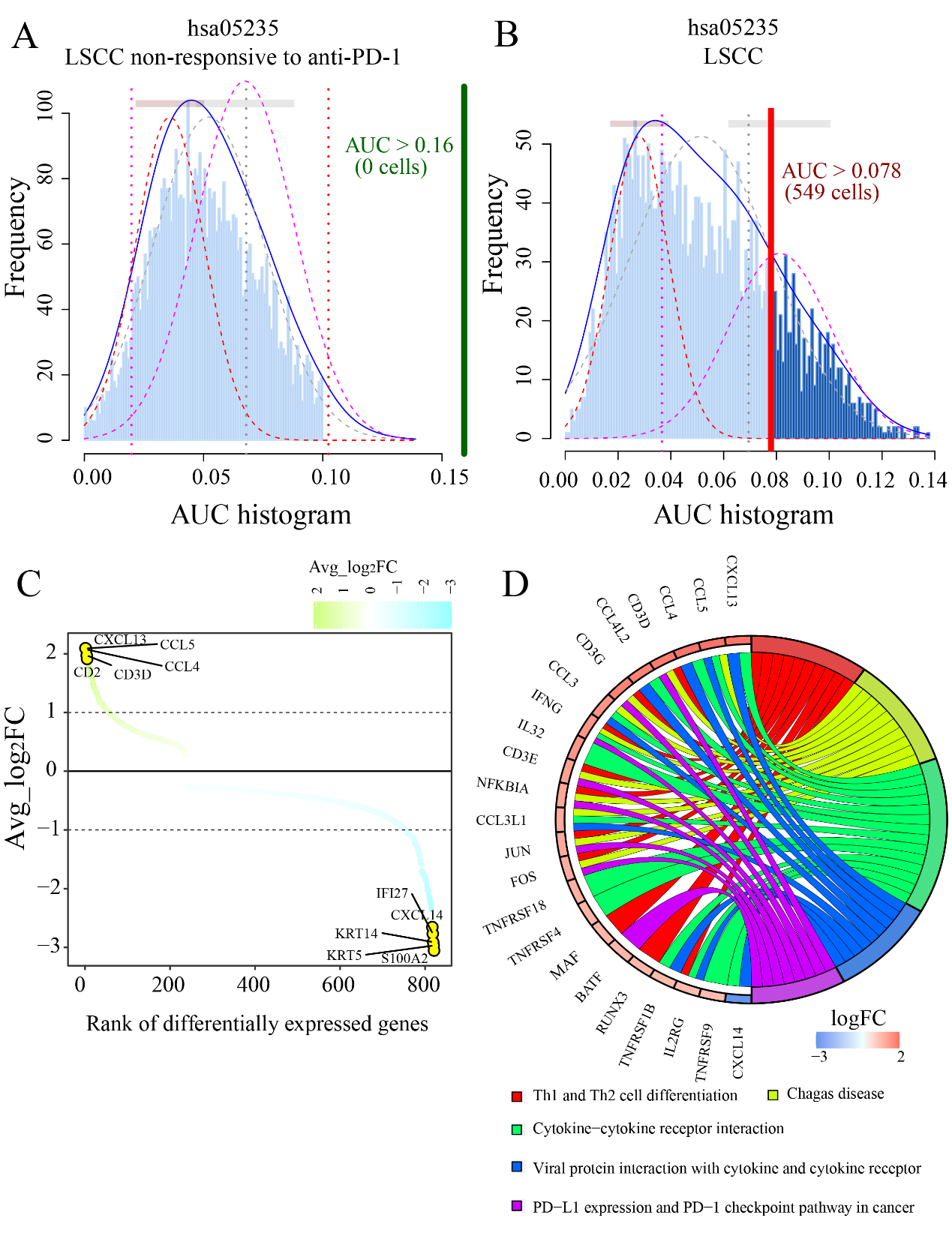


Figure S6: Putative immune checkpoint therapy-associated gene signatures in laryngeal squamous cell carcinoma single cells

(A, B) The activity of PD-L1 expression and PD-1 checkpoint pathway were calculated in wild type and anti-PD1 non-responsive laryngeal squamous cell carcinoma (LSCC) single cells using AUCell. (C) Potential gene signatures associated with immune checkpoint therapy were identified from LSCC single cells. (D) Kyoto encyclopedia of genes and genomes pathway enrichment analysis of such signatures. These findings provide preliminary evidence regarding putative immune checkpoint therapy-associated gene signatures in LSCC single cells, suggesting potential targets and pathways that could be explored for therapeutic interventions.


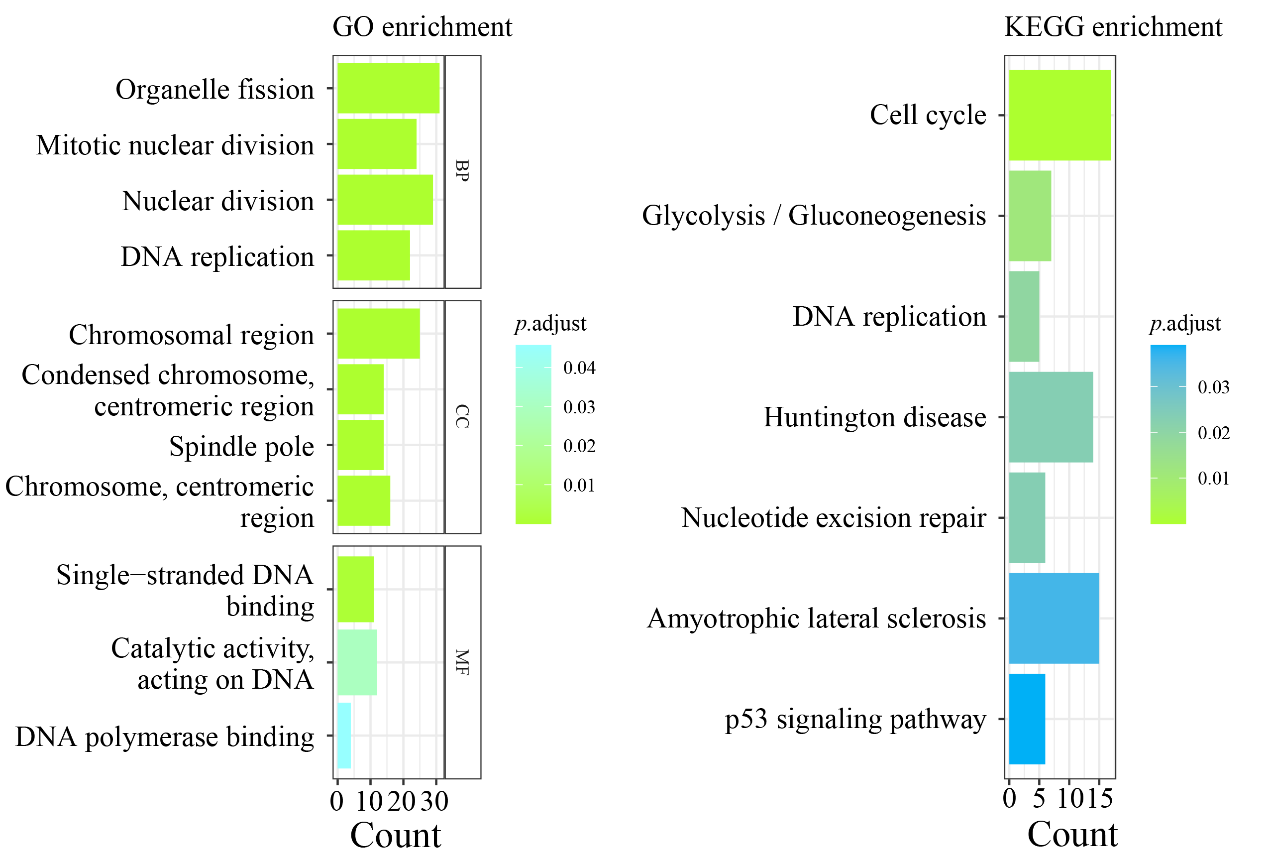


Figure S7: Putative molecular mechanisms of triosephosphate isomerase 1 co-expressed genes in laryngeal squamous cell carcinoma tissue


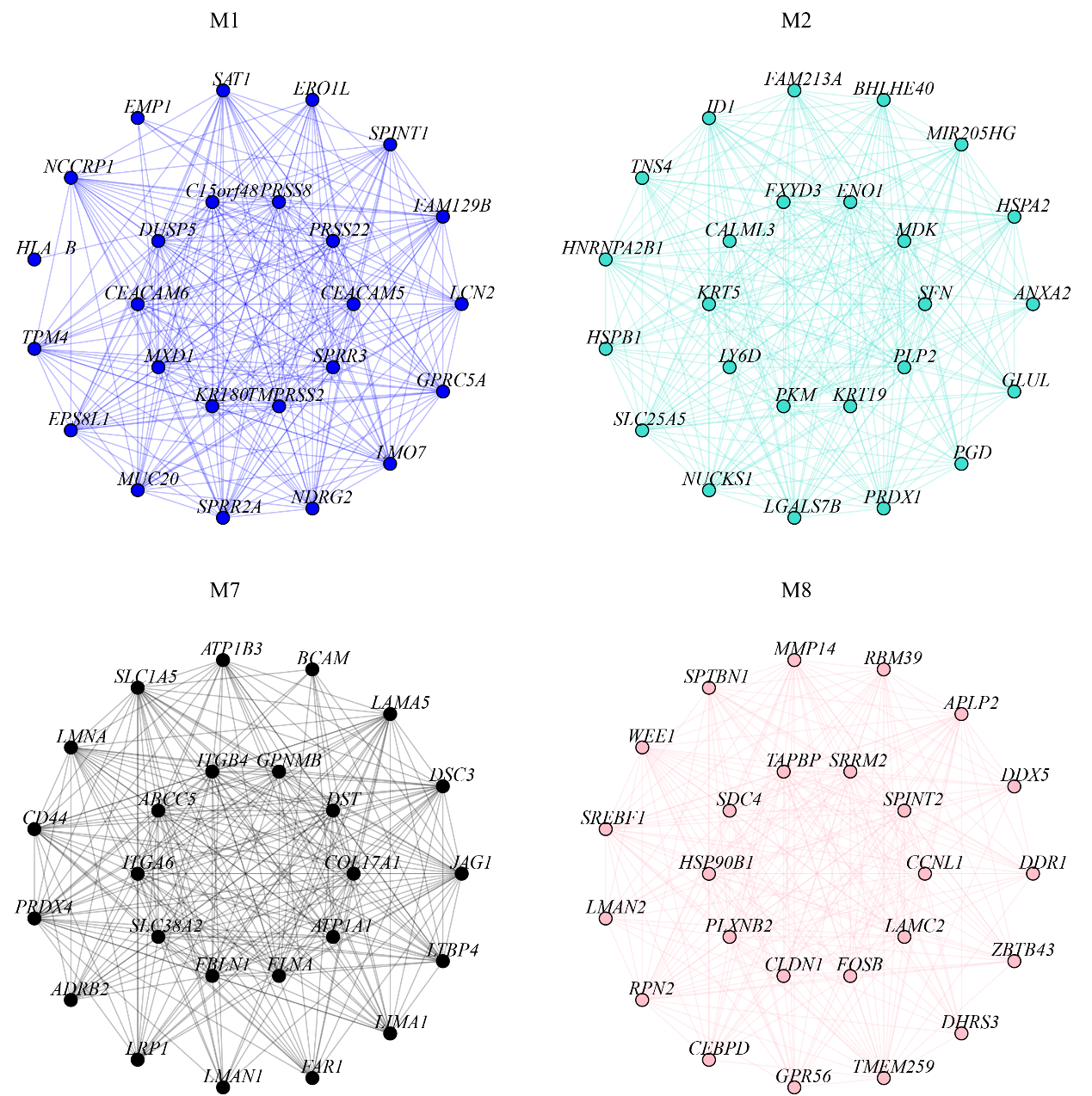


Figure S8: Hub genes of laryngeal squamous cell carcinoma specific co-expressed gene modules

A total of four laryngeal squamous cell carcinoma specific gene modules were identified, including M1, M2, M7, and M8. Hub genes
